# Digitally Guided Tooth Autotransplantation: A Systematic Review of Clinical, Radiographic and Operative Outcomes

**DOI:** 10.3390/jcm15218601

**Published:** 2026-11-04

**Authors:** Abdullah Saleh Almutairi, Osama Mansour Alnoodaly, Sulaiman Mohammed Alrasheed, Salman Tarique Ansari, Shaul Hameed Kolarkodi

**Affiliations:** 1 Department of Periodontology and Implant Dentistry, College of Dentistry, Qassim University, Buraidah 52571, Qassim, Saudi Arabia; dr.almutairi@qu.edu.sa (A.S.A.); s.ansari@qu.edu.sa (S.T.A.); 2 Medical City, College of Dentistry, Qassim University, Buraidah 52571, Qassim, Saudi Arabia; ososzz2002@gmail.com (O.M.A.); sulaiman.dent@gmail.com (S.M.A.); 3 Department of Oral and Maxillofacial Diagnostic Sciences, College of Dentistry, Qassim University, Buraidah 52571, Qassim, Saudi Arabia

**Keywords:** tooth autotransplantation, cone beam computed tomography, three-dimensional printing, surgical guide, computer-assisted surgery, systematic review

## Abstract

**Background/Objectives:** Digital guidance of tooth autotransplantation, using cone beam computed tomography (CBCT)-based planning with three-dimensionally (3D) printed donor tooth replicas, surgical guides, navigation or robotics, aims to shorten extra-alveolar time and improve positioning. This systematic review synthesised the clinical, radiographic and operative outcomes of digitally guided autotransplantation and compared them with conventional technique. **Methods:** Following PRISMA 2020 and PROSPERO registration CRD420261434791, MEDLINE (PubMed and Ovid), Embase, Scopus, Web of Science, CENTRAL and ClinicalTrials.gov were searched from inception to August 2026, with Google Scholar and reference-list screening. Clinical studies of at least five digitally guided transplanted teeth or patients were eligible. Two reviewers independently screened, extracted data and assessed risk of bias (RoB 2, ROBINS-I or the JBI case-series checklist); certainty was rated with GRADE. **Results:** Twenty-five studies reported in 33 publications (2012–2026; one randomised, eight comparative non-randomised, sixteen single-arm; approximately 1300 patients) were included. All used CBCT- or CT-derived 3D-printed replicas, eleven with printed guides; none used navigation or robotics. In guided arms, survival was 92–100% and success 86–100% at 6 months to 5 years. Extra-alveolar time was under one minute in eleven reports, most donor teeth fitted at the first attempt, and positional deviation from the plan was about 1–2 mm. Compared with the conventional technique, guidance shortened surgical time by 13–15 min in two studies (low certainty) and was associated with higher survival and success and less root resorption in retrospective cohorts at serious risk of bias, whereas the registered controlled trial and a prospective paediatric cohort found no significant clinical difference (very low certainty). **Conclusions:** Digital guidance improves the operative efficiency and positional control of autotransplantation. Clinical superiority over conventional technique remains unproven; randomised trials with standardised outcomes and follow-up beyond two years are needed.

## 1. Introduction

Tooth autotransplantation is the surgical transfer of a tooth from one site to another in the same individual. It offers a biological alternative to implants and fixed prostheses, particularly for young patients in whom implant placement must be deferred until growth is complete, because a successfully transplanted tooth retains a periodontal ligament, permits continued alveolar development and can be moved orthodontically [1,2,3]. Long-term series have reported survival above 90% for premolars transplanted with immature roots [2], and acceptable outcomes for mature third molars provided that root canal treatment is completed in time [4,5]. Recent syntheses continue to report pooled survival of about 87% to 96% and success of about 84% to 92% across indications [6,7,8,9], with survival of about 94% and success of about 85% when immature donors are transplanted in children and adolescents [10], and interest in the procedure has grown with advances in imaging, planning and regenerative adjuncts [11,12,13].

The success of an autotransplant depends on conditions at three levels. General prerequisites include a patient who is healthy enough for oral surgery, is motivated, maintains adequate oral hygiene and can attend for follow-up; and systemic disease that impairs healing and heavy smoking are relative contraindications [1,12]. Local prerequisites concern the donor tooth, the recipient site and the surgery itself. The donor should be extractable without fracture or damage to its periodontal ligament, which favours single or conical roots and an open, unerupted or easily luxated tooth; root development between one-half and three-quarters of the final length is the most favourable stage, because it allows pulp revascularisation and continued root growth, whereas a mature donor requires root canal treatment within a few weeks of surgery [1,2,3,4]. The recipient site should offer enough bone in all dimensions to receive the root without an exposed root surface, enough mesiodistal and interocclusal space for the crown, and no acute infection [1,14]. Surgically, the periodontal ligament must be preserved by atraumatic extraction, a short extra-alveolar time, minimal handling of the root, a passively fitting socket, slight infraocclusal placement and flexible splinting for a short period [1,15,16]. The teeth most commonly suitable as donors are premolars, usually removed for orthodontic reasons and transplanted to the anterior maxilla or to sites of agenesis in growing patients; third molars, transplanted to first- or second-molar sites in adolescents and adults; and, less often, impacted canines and supernumerary teeth [2,3,7,16].

The prognosis of a transplanted tooth therefore depends on the preservation of the periodontal ligament cells on the donor root surface. Two operative variables are widely regarded as critical: the time the donor tooth spends outside the alveolus and the number of times it is trial-fitted into the recipient socket [1,15,17]. Each additional fitting attempt exposes the root surface to mechanical trauma and desiccation, and trauma to the periodontal ligament is the explanatory factor for replacement resorption and ankylosis [15,16]. Conventional technique requires the surgeon to extract the donor tooth before the recipient socket can be shaped to it, so that socket preparation and trial fitting take place while the tooth is out of the mouth.

Digital technologies have been introduced to break this dependency. Since the first reports of computer-aided rapid prototyping of donor tooth models in the early 2000s [17,18], CBCT segmentation and 3D printing have made it possible to fabricate a dimensionally accurate replica of the donor tooth before surgery. The replica is used to prepare and check the recipient socket while the donor tooth remains in its own alveolus, so that the tooth can be transferred in a single movement [17,19]. More recent workflows add virtual planning of the transplant position, 3D-printed static guides that constrain osteotomy position and depth, and, in individual reports, dynamic navigation or robotic assistance [20,21]. Automated segmentation with artificial intelligence has further shortened the planning stage [22], and population-derived “universal” donor models have been proposed to avoid patient-specific printing altogether [23].

The clinical rationale is that these tools shorten extra-alveolar time, reduce the number of fitting attempts and place the tooth closer to its planned position, and that these operative gains translate into better periodontal healing, fewer complications and higher survival. Whether the second part of that chain has been demonstrated is less clear. The primary studies are heterogeneous: they range from small single-arm series describing a new workflow to retrospective and prospective comparisons against conventional technique, they use different definitions of success, and they report follow-up ranging from an immediate postoperative scan to several years. Previous systematic and umbrella reviews of autotransplantation have concentrated on prognostic factors, the root development stage and overall survival rather than on the contribution of digital guidance [6,14,16,24]. Reviews that address digital adjuncts have either been narrative overviews of technique [12,25] or have examined complementary technologies for a single indication [26]; a design-stratified, risk-of-bias-appraised synthesis of the clinical studies of digitally guided autotransplantation is lacking.

The aim of this systematic review was therefore to identify and critically appraise clinical studies of digitally guided tooth autotransplantation and to synthesise their clinical, radiographic and operative outcomes. The specific questions were, first, what survival, success, complication and operative outcomes are reported after digitally guided autotransplantation; second, how accurately the achieved tooth position matches the virtual plan; and third, whether comparative studies show a difference in these outcomes between digitally guided and conventional freehand technique. The review adds to previous work by separating comparative from single-arm evidence, appraising each with a design-appropriate tool, distinguishing the operative effects of guidance from its effects on the prognosis of the transplanted tooth, and rating the certainty of the comparative evidence rather than pooling survival across designs.

## 2. Materials and Methods

### 2.1. Protocol and Reporting

The review was conducted and reported in accordance with the Preferred Reporting Items for Systematic Reviews and Meta-Analyses (PRISMA) 2020 statement [27] and the PRISMA-S extension for reporting literature searches [28]. The protocol was registered prospectively in PROSPERO CRD420261434791 on 27 June 2026; no deviations from the registered protocol occurred.

### 2.2. Eligibility Criteria

Eligibility was defined using the population, intervention, comparator, outcome and study design (PICOS) framework.

Population. Patients of any age undergoing autotransplantation of a tooth, of any type and at any stage of root development, to a recipient site in the same individual.

Intervention. Autotransplantation in which at least one stage of the procedure was digitally guided. Digital guidance was defined as the use of CBCT- or CT-based three-dimensional imaging together with one or more of the following: a 3D-printed (rapid-prototyped) replica of the donor tooth used to prepare or check the recipient socket; computer-aided virtual planning of the transplant position; a static 3D-printed surgical guide or template; dynamic (real-time) navigation; or robotic assistance. Studies in which CBCT was used for diagnosis only, without a replica, guide, navigation or robotic component in the surgical workflow, were not considered digitally guided.

Comparator. Conventional autotransplantation without digital guidance, an alternative digital workflow, or no comparator.

Outcomes. At least one clinical, radiographic or operative outcome of the transplanted tooth: survival; success according to stated criteria; periodontal healing; root resorption; ankylosis; pulp status or need for endodontic treatment; positional accuracy relative to the virtual plan; extra-alveolar (extra-oral) time; number of fitting attempts; or operative time.

Study design. Randomised and non-randomised controlled trials, prospective and retrospective cohort studies, and case series. To exclude anecdotal reports, a study had to report outcomes for at least five digitally guided transplanted teeth or patients. Case reports and case series below this threshold, reviews, in vitro, cadaver and purely computational studies, technical notes without patient outcomes, retracted publications, and studies of procedures other than tooth autotransplantation (for example, intentional replantation or implant placement) were excluded. For studies reporting clinical or radiographic outcomes, a minimum follow-up of six months was required; this requirement was not applied to studies reporting only operative or accuracy outcomes measured at surgery or on the immediate postoperative scan. No restriction was placed on publication year. Records were limited to English-language publications.

### 2.3. Information Sources

A systematic search was conducted from 1 August 2026 to 15 August 2026 in MEDLINE via PubMed, MEDLINE via Ovid, Embase via Ovid, Scopus, Web of Science Core Collection and the Cochrane Central Register of Controlled Trials (CENTRAL) via the Cochrane Library. ClinicalTrials.gov was searched to identify registered, ongoing or unpublished trials. Google Scholar was searched as a supplementary source, and the first 200 results were screened. The reference lists of included articles and of relevant systematic reviews were screened by hand. Each database was searched from inception to the search date.

### 2.4. Search Strategy

The search strategy was built around three concept blocks: tooth and dental terminology; autotransplantation terminology; and digital guidance and computer-assisted surgical technologies, including CBCT, 3D-printed donor tooth replicas, CAD/CAM, static surgical guides, dynamic navigation and robotic assistance. Within each block, Medical Subject Headings (MeSH), Emtree terms where applicable and free-text synonyms were combined with OR, and the three blocks were combined with AND. No outcome block was included, because restricting the search to outcome terms could have reduced sensitivity for studies that report the outcomes of interest only in the full text. The MEDLINE (PubMed) strategy served as the master strategy and was translated for the other platforms, with syntax, truncation, phrase searching and field tags adapted to each database. An English-language limit was applied in PubMed, and conference abstracts and conference reviews were excluded from the Embase result set; no other database limits were applied. The full strategies and the number of records retrieved from each source are provided in Supplementary Tables S1–S8.

### 2.5. Selection Process

Records from all sources were exported to EndNote (version 21; Clarivate Plc, Philadelphia, PA, USA) and duplicates were removed using its automatic duplicate detection followed by manual verification of every flagged pair. Titles and abstracts, and subsequently full texts, were screened independently and in duplicate by two reviewers against the eligibility criteria in Rayyan (Rayyan Systems Inc., Cambridge, MA, USA), with decisions blinded between reviewers until both had completed each stage; the reviewers were calibrated on a pilot sample of 50 records before formal screening began. Disagreements were resolved by discussion and, where necessary, by a third reviewer. Inter-rater agreement at the title-and-abstract stage was 0.80. The date, route and outcome of every retrieval attempt are documented for each report in Supplementary Table S10. Reports that could not be obtained through any of these routes were recorded as not retrieved, with the reason included. Where several publications reported the same cohort or trial, they were linked and treated as one study with multiple reports (PRISMA 2020 item 16a), with one publication designated the primary report for each outcome and the others used only for data not duplicated in it. No automation tool or machine learning classifier was used at any stage. Reasons for exclusion at full-text assessment are reported in the PRISMA flow diagram and in Supplementary Table S9.

### 2.6. Data Collection

Data were extracted independently by two reviewers into a piloted extraction form and reconciled by consensus. The following items were extracted for each study: bibliographic details; country and setting; study design and comparator; guidance modality and the planning software and printing hardware used; number of patients and transplanted teeth; age; donor tooth type and recipient site; apex maturity; minimum and mean follow-up; primary and secondary outcomes as defined by the authors; main results; and funding and conflict-of-interest statements. Where a paper reported inconsistent values for the same quantity, both values were recorded and are reported. Corresponding authors were contacted for missing or unclear data.

### 2.7. Outcomes

The primary outcomes of the review were transplant survival (the tooth remaining in situ at the last follow-up) and success according to each study’s stated clinical and radiographic criteria. Secondary outcomes were root resorption, ankylosis, need for endodontic treatment, periodontal parameters (probing depth, bleeding on probing, mobility), positional accuracy of the transplanted tooth relative to the virtual plan, extra-alveolar time, number of fitting attempts, and operative time. Outcome definitions were recorded as reported by the authors and were not re-classified.

### 2.8. Risk of Bias Assessment

Risk of bias was assessed independently by two reviewers, with disagreements resolved by consensus, using tools matched to study design. The one study that reported random allocation was assessed with the Cochrane risk-of-bias tool for randomised trials (RoB 2) [29], which addresses five domains (randomisation process, deviations from intended interventions, missing outcome data, measurement of the outcome and selection of the reported result) and yields an overall judgement of low risk, some concerns or high risk. Comparative non-randomised studies were assessed with the Risk Of Bias In Non-randomised Studies of Interventions (ROBINS-I) tool [30], which addresses seven domains (confounding, selection of participants, classification of interventions, deviations from intended interventions, missing data, measurement of outcomes and selection of the reported result) and yields an overall judgement of low, moderate, serious or critical risk of bias, taken as the most severe domain-level judgement. A study described by its authors as randomised but whose methods stated that no random allocation sequence was generated was assessed with ROBINS-I, and the discrepancy is reported. For each comparative study the target comparison was specified in advance; where the comparison did not contrast digital guidance with conventional technique (for example, a comparison of two digital workflows or of an adjunctive biomaterial within replica-guided surgery), this was recorded and the study was not treated as evidence on the effect of guidance itself. Single-arm studies, and studies whose internal comparison was not an intervention comparison, were assessed with the JBI Critical Appraisal Checklist for Case Series [31], which comprises ten items answered yes, no, unclear or not applicable; item-level responses are reported rather than a summary score, and an overall appraisal was formed with greatest weight given to consecutive and complete inclusion of participants and to completeness of outcome reporting. Results are presented as a traffic-light plot in the main text and as summary and domain-level tables in the Supplementary Material.

### 2.9. Synthesis Methods and Certainty of Evidence

Studies were grouped by design (randomised, comparative non-randomised or single-arm) and by guidance modality (replica only; replica with static guide; alternative digital workflow). A quantitative synthesis was prespecified if at least three comparative studies reported the same outcome, measured in the same way, at comparable follow-up. This condition was not met: the comparative studies differed in comparator, outcome definitions, follow-up and analysis, and the single-arm studies did not permit the estimation of a comparative effect. Findings were therefore synthesised narratively following the Synthesis Without Meta-analysis (SWiM) reporting guideline [32], with outcomes tabulated by study and the direction of effect summarised for each comparative study, structured according to the SWiM items (grouping, standardised metric, synthesis method, presentation, and limitations of the synthesis). Internal inconsistencies within the reports are noted in the results tables rather than resolved by assumption. Where reports from the same centre covered overlapping periods, patients were counted once in the totals and the overlap is stated.

The certainty of the evidence for each outcome of the comparison between digitally guided and conventional autotransplantation, and separately for comparisons between digital workflows, was rated with the GRADE approach [33]. For outcomes informed only by non-randomised studies the certainty started at low; for the outcome informed by the randomised trial it started at high. Certainty was downgraded for serious risk of bias, inconsistency, indirectness or imprecision, and considered for upgrading where a large effect was observed in the absence of plausible confounding. Single-arm studies contributed to the description of outcomes but not to the certainty rating of comparative effects. Ratings were made by the same two reviewers independently and agreed by consensus, and are presented in a summary-of-findings table.

## 3. Results

### 3.1. Study Selection

**Figure 1:**
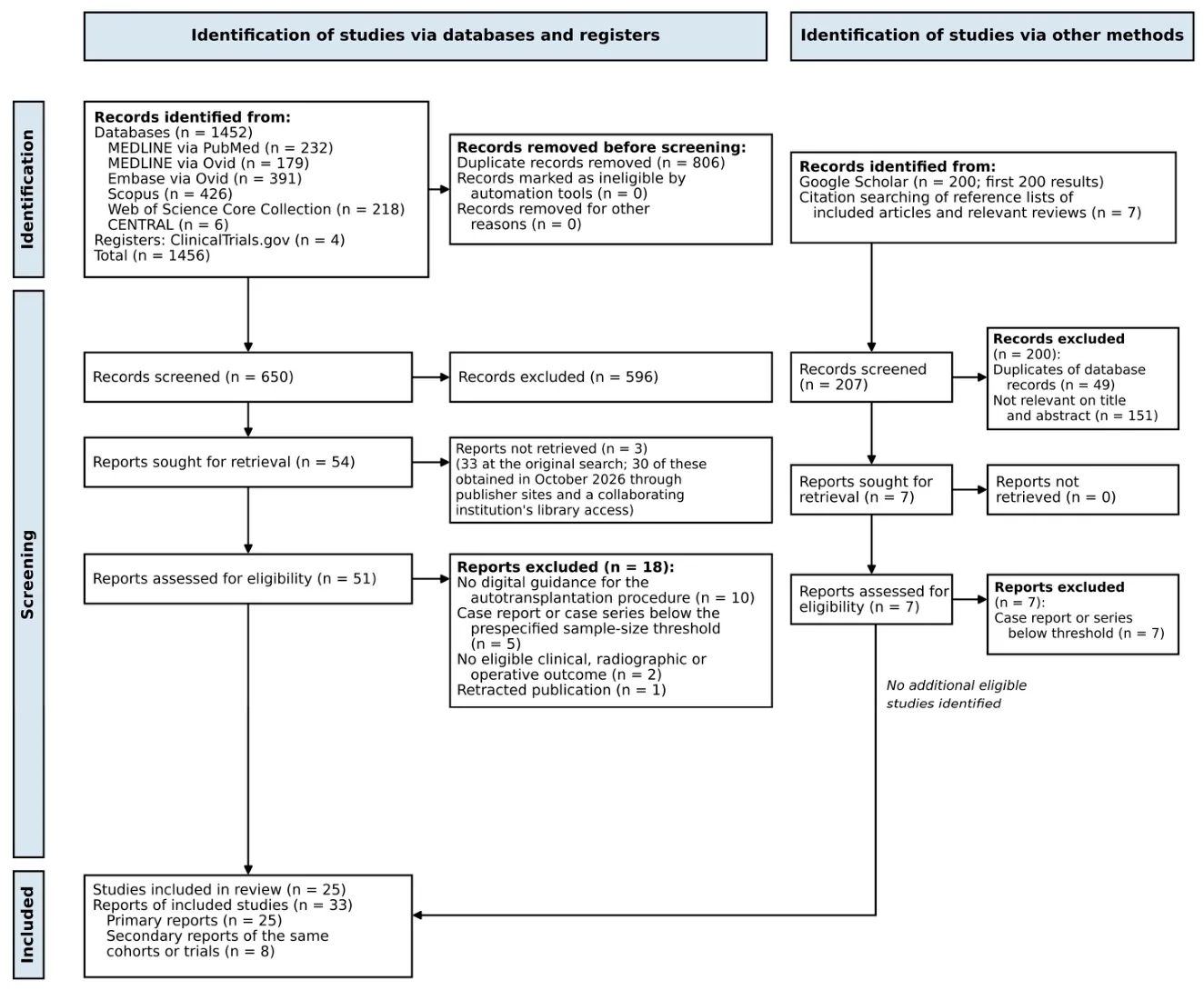
PRISMA 2020 flow diagram of study selection. Databases and registers were searched from inception to August 2026; full texts not obtained at that time were sought again in October 2026 through additional routes (Section 2.5). No automation tool was used at any stage of the selection process.

The database and registry searches identified 1456 records, of which 806 were duplicates. After title and abstract screening of 650 records, 54 reports were sought for retrieval. At the time of the original search, 33 of these could not be obtained; after the additional retrieval routes described in Section 2.5, 30 of the 33 were obtained in full text, so that 51 reports were assessed for eligibility and three could not be retrieved (two reports in a journal not available through any of the libraries accessed, and one report in a subscription journal whose abstract describes an en bloc technique without digital guidance; Supplementary Table S10). Eighteen reports were excluded: ten because the surgical workflow contained no digital guidance component, five because they were case reports or series below the prespecified sample-size threshold, two because they reported no eligible clinical, radiographic or operative outcome, and one because it had been retracted (Supplementary Table S9). Screening of the first 200 Google Scholar results identified 49 duplicates of database records and 151 irrelevant records. Reference-list checking of the included reports identified seven further candidate reports, all of which were case reports or series of fewer than five cases and were excluded. Thirty-three reports describing 25 studies were included: 25 primary reports [18,20,22,23,34,35,36,37,38,39,40,41,42,43,44,45,46,47,48,49,50,51,52,53,54] and eight secondary reports of the same cohorts or trials [4,17,19,55,56,57,58,59]. The selection process is summarised in Figure 1.

### 3.2. Characteristics of the Included Studies

The characteristics of the included studies are presented in Table 1, with the linked secondary reports listed beneath their primary study. The primary reports were published between 2012 and 2026, and 18 of the 25 appeared in or after 2021; the secondary reports extend the earliest cohort back to 2001. One study was a randomised comparison of a patient-specific model with a generic donor tooth model [23]. Eight studies were comparative but non-randomised: five compared a digitally guided workflow with conventional technique [38,39,41,45,53], two compared a fully guided workflow with a replica alone [22,44], and one compared an adjunctive biomaterial (concentrated growth factor) between two replica-guided arms [36]. One of these was described in its title as a randomised trial, but its appendix states that no random allocation sequence was generated and that the operator assigned participants [44]; it was therefore treated as non-randomised. Four comparative studies were prospectively registered [22,41,44,53]. The remaining sixteen studies were single-arm series or cohorts [18,20,34,35,37,40,42,43,46,47,48,49,50,51,52,54]. Studies were conducted in China (ten), Spain (three), and Italy, France, Latvia, Taiwan, South Korea, Turkey, the Netherlands, Belgium and Australia (one each); three studies did not state the country of the clinical setting [36,39,42]. Together, the primary reports enrolled approximately 1300 patients, with individual samples ranging from five to 182 patients; reports from the same centre with overlapping recruitment periods (Hangzhou, Leuven, Leiden, Barcelona, Seoul and Fuzhou) were counted once. Donor teeth were third molars in sixteen studies; premolars or canines predominated in six studies, four of which transplanted teeth in children or adolescents [39,40,42,51,53,54], two included mixed donor types [20,47], and one did not tabulate donor type [52]. Apex maturity followed donor type: the third-molar series transplanted mature teeth with closed apices, with the exception of the immature impacted third molars of Lejnieks et al. and Erdem and Gümüşer [41,50], and the paediatric series transplanted immature teeth. Follow-up ranged from an immediate postoperative scan with no clinical follow-up [18,22,42,51] to a mean of 5.2 years [45]; the long-term reports were the retrospective cohorts of Xia et al. (mean 5.2 years) [45], EzEldeen et al. (mean 4.5 years) [53] and Ainiwaer et al. (mean 4 years) [48], and, among the secondary reports, the 12-year survival analysis of the Seoul cohort [4].

### 3.3. Digital Workflows

All 25 studies used a CBCT- or CT-derived, three-dimensionally printed replica of the donor tooth to prepare or verify the recipient socket before extraction of the donor tooth (Supplementary Table S11); in the randomised study, one arm used a generic replica selected from twelve population-derived variants rather than a patient-specific model [23]. Eleven studies combined the replica with a 3D-printed or milled surgical guide or template that constrained the position or depth of the osteotomy [20,22,34,37,43,44,46,47,52,53,54]; one of these added a printed occlusal splint and individual arch bar for fixation [34], and one used a trephine-based preparation guide with separate position and fixation guides [44]. Two of the most recent studies used automated, artificial intelligence-based segmentation of the CBCT data [22,43]. Replica materials included photopolymer resins, cobalt-chromium alloy [42,50], titanium [19] and, in the earliest studies, starch- or resin-based rapid-prototyping materials [18,58]. No included study used dynamic navigation or robotic assistance; the one robot-assisted report identified by the search was a single-patient proof of concept and was excluded [21]. Planning software was reported in 21 studies; the most frequently named packages were Mimics and 3-matic (Materialise), ProPlan CMF (Materialise), 3D Slicer with the DentalSegmentator extension, SimPlant, Blue Sky Plan and Meshmixer.

### 3.4. Clinical and Radiographic Outcomes

Clinical and radiographic outcomes are summarised in Table 2. Survival of digitally guided transplants was reported numerically in fourteen studies. At six months to two years it ranged from 95.5% (21 of 22 transplants at a median of 23 months) [40] to 100% [20,34,38,41,46,50,52,54]. At longer follow-up it was 97.2% at a mean of 29 months [49], 95.8% at a mean of 4 years [48], 99% for replica-guided third molars at a mean of 5.2 years [45] and 92% for CBCT-guided premolars in children at a mean of 4.5 years [53]; two further studies stated that all transplants remained in function without giving a rate [35,43]. The secondary reports from the Seoul cohort show the attrition that longer observation reveals: a failure rate of 4.5% over 2 to 60 months [58], and cumulative survival of 88.1% at 3 years and 68.2% at 12 years for mature-tooth transplants performed with rapid-prototyped models between 2001 and 2010 [4]. Success, defined by clinical and radiographic criteria that differed between studies, ranged from 86% at 4.5 years [53] and 87.5% (7 of 8 teeth, mean follow-up 2 years) [34] to 100% at 1 to 2 years [20,35,50]; intermediate values were 90% [39], 90.5% [40], 91.2% [46], 91.7% [49], 92.3% to 100% [36], 95.8% [48] and 98% [45]. Root resorption was reported as absent in eight series [34,35,40,43,46,50,52,54], as a single case in four [20,36,37,49], as 13% of replica-guided versus 36% of conventional transplants at 12 months in the controlled trial by Lejnieks et al. (*p* = 0.057) [41], and as inflammatory resorption in 1% of replica-guided versus 41% of unguided mature third molars over 5 years by Xia et al. (*p* < 0.001) [45]. Ankylosis was reported in one of eight teeth by He et al. [34], in one of 36 by Lucas-Taulé et al. [49], and in none of the replica-guided teeth in the other studies that assessed it; Kizek et al. observed radiological signs of ankylosis in three of 14 conventionally treated patients and in none of the 10 replica-guided patients [39], and in the Leuven case–control study replacement resorption occurred in one guided and four conventional transplants [57]. Endodontic treatment was performed prophylactically in all mature donors in seven studies [18,34,35,45,46,49,52], was required in two of 10 teeth by Salviati et al. [43], and was needed by 50% to 59% of patients in the rapid-prototyping group versus 92.3% in the conventional group in the retrospective cohort by Hwang et al. [38]; in the paediatric series no transplant required endodontic treatment [50,54]. Periodontal parameters, where reported, were within physiological limits at the final follow-up in all series, with transient probing depths above 3 mm in the first weeks after surgery in two studies [35,37], and Pedrinaci et al. reported a gain in clinical attachment of 2.8 mm relative to the hopeless teeth that the transplants replaced [46].

### 3.5. Operative and Accuracy Outcomes

**Figure 2:**
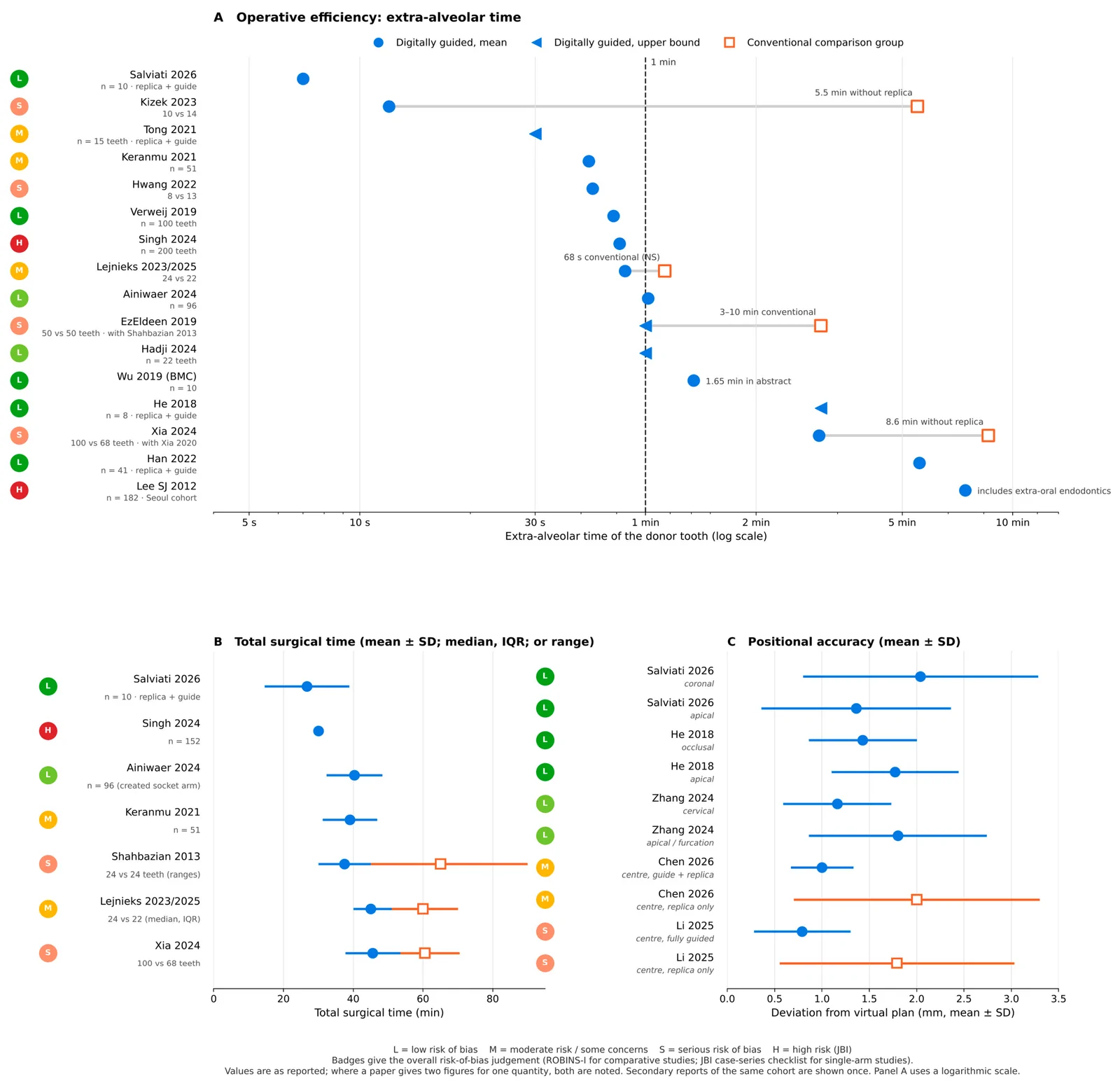
Operative outcomes of digitally guided autotransplantation reported in the included studies. (**A**) Extra-alveolar time of the donor tooth, on a logarithmic scale; the dashed vertical line marks 1 min. Filled blue circles show the value reported for the digitally guided group (mean unless stated), and blue triangles show an upper bound where only a bound was reported (for example, “under 1 min”). Open orange squares show the conventional comparison group where one was reported, joined to the guided value by a grey line. Data are from Salviati et al. [43], Kizek et al. [39], Tong et al. [54], Keranmu et al. [36], Hwang et al. [38], Verweij et al. [51], Singh et al. [42], Lejnieks et al. [41,55], Ainiwaer et al. [48], EzEldeen et al. with Shahbazian et al. [53,57], Hadji and Bader [40], Wu et al. [35], He et al. [34], Xia et al. [45,56], Han et al. [37] and the Seoul cohort [17,18,58,59]. (**B**) Total surgical time. Circles with horizontal lines show the mean ± SD, median and IQR, or range, as reported, for the guided group (blue); open orange squares show the conventional group [36,41,42,43,45,48,57]. (**C**) Deviation of the transplanted tooth from the virtual plan (mm, mean ± SD) at the reported measurement points. Blue shows the digitally guided workflow; orange shows the replica-only comparison workflow in the two comparative studies [22,34,43,44,47]. Coloured badges give each study’s overall risk-of-bias judgement (RoB 2 or ROBINS-I for comparative studies; JBI case-series checklist for single-arm studies): dark green (L), low; light green (L), low to moderate; yellow (M), moderate or some concerns; orange (S), serious; red (H), high. Values are as reported and mix means, medians and upper bounds. Secondary reports of the same cohort are shown once. See Supplementary Table S12.

Operative outcomes are presented in Supplementary Table S12 and Figure 2. Extra-alveolar time was reported in 22 reports. In eleven reports of replica-guided socket preparation the mean, median or upper bound was below one minute: 7 s [43], 12 s [39], under 30 s in all cases [54], 42 s [36], 43 s [38], 49 s (range 1 to 600 s) in the multicentre series of 100 transplants by Verweij et al. [51], 51 s [42], 53 s in the controlled trial by Lejnieks et al. [55], 61 s [48], under one minute in all five cases of the Leiden proof of concept [19] and under one minute in the Leuven paediatric cohort [53,57]. Times of one to three minutes were reported by Hadji et al. (under one minute with two outliers of 3 and 5 min) [40], Wu et al. (mean 1.35 min; 1.65 min in the abstract) [35], He et al. (less than 3 min in all cases) [34], and Xia et al. (2.5 min in 28 cases and 2.96 min in 100 replica-guided cases) [45,56]. Longer times were reported by Han et al. (mean 5.56 min), whose protocol included seating trials with the donor tooth itself [37], and by the Seoul group, whose early CT-based models were combined with extra-oral endodontic treatment in some cases (means of 6.7, 7.4 and 7.6 min) [17,18,58,59]. The number of fitting attempts was reported in eleven reports: an immediate fit without additional attempts was achieved in 82% of 100 transplants by Verweij et al. [51], in 80% of 200 transplants by Singh et al. [42], in six of eight teeth by He et al. [34] and in all transplants in three small series [19,52,54]; Han et al. reported a mean of 2.61 positioning trials [37] and Ainiwaer et al. 2.43 [48]; Lejnieks et al. reported a single fitting in 63% of replica-guided versus 32% of conventional procedures, a difference that was not statistically significant in the trial’s primary operative report [41,55]; Shahbazian et al. reported zero to three attempts with the replica versus four to seven without [57]; and in the two comparisons of digital workflows a fully guided system did not reduce attempts relative to a replica alone (1.07 versus 1.13) [44], whereas a patient-specific replica required fewer re-preparations of the socket than a generic replica (*p* = 0.025 and 0.042 for maxillary and mandibular sites) [23]. Total surgical time, reported in nine reports, ranged from 26.7 min [43] to 45.6 min [45] for guided procedures; in the three studies with a conventional arm it was 45 versus 60 min (median) in the controlled trial [41], 45.6 versus 60.5 min in the Hangzhou cohort [45], and 30 to 45 versus 40 to 90 min in the Leuven case–control study [57].

Positional accuracy relative to the virtual plan was assessed in six studies. Salviati et al. reported a mean surface deviation of 0.63 ± 0.45 mm and linear displacements of 2.04 ± 1.24 mm coronally and 1.36 ± 1.00 mm apically at 18 months [43]. He et al. reported deviations of 1.43 ± 0.57 mm at the occlusal point and 1.77 ± 0.67 mm at the apex immediately after surgery, with a further 0.66 to 0.67 mm of settling before stability was reached at 3 to 6 months [34]. Zhang et al., in 50 transplants placed with a replica and positional guide, reported deviations of 1.16 ± 0.57 mm at the cervix, 1.80 ± 0.94 mm at the apex or furcation and 6.82 ± 3.14° in the long axis, with larger apical deviation for multi-rooted donors [47]; Wu et al. reported a 99% overlap between the planned and the achieved tooth volume in five cases [52]. In the two comparisons of digital workflows, a dual-template guided arm achieved a centre deviation of 1.00 ± 0.33 mm and a buccolingual angular deviation of 2.76 ± 2.31° compared with 2.00 ± 1.30 mm and 8.61 ± 4.55° in the replica-only freehand arm (*p* = 0.023 and *p* = 0.001) [22], and a fully guided system achieved a centre deviation of 0.79 ± 0.51 mm and a long-axis deviation of 3.87 ± 2.93° compared with 1.79 ± 1.24 mm and 12.98 ± 7.83° with a replica alone (*p* = 0.006 and *p* < 0.001) [44].

### 3.6. Findings of the Comparative Studies

Five studies, reported in six publications, compared digitally guided autotransplantation with conventional autotransplantation. In the non-randomised controlled trial by Lejnieks et al., survival at one year was 100% in both arms; mobility, bleeding on probing and periodontal pockets at 12 months were less frequent in the replica-guided arm (0% versus 13.6%, 4.1% versus 13.6% and 4.1% versus 13.6%, respectively), but in the adjusted mixed models, group assignment was not a significant predictor of these outcomes, whereas operative time, Moorrees stage and the number of donor placement attempts were [41]; in the same trial, replicas reduced total surgical time by an adjusted 13.4 min (95% confidence interval 2.3 to 24.5) but did not significantly reduce extra-alveolar time (52.7 versus 67.7 s; adjusted difference −9.4 s, 95% confidence interval −40.9 to 22.2) [55]. In the prospective paediatric cohort of EzEldeen et al., survival and success at a mean of 4.5 years were 92% and 86% with CBCT guidance versus 84% and 78% with conventional planning, differences the authors reported as not significant [53]; the earlier case–control report from the same unit found fewer fitting attempts, shorter extra-alveolar and surgical times, and two failures with the replica versus six without, without a statistical test [57]. In the retrospective cohort of Xia et al., replica use was independently associated with success, survival and freedom from inflammatory resorption over a mean of 5.2 years (adjusted hazard ratios for the absence of a replica of 23.3, 13.3 and 31.8, respectively, with wide confidence intervals) and with shorter surgical and extra-alveolar times, but replica use was also associated with other changes in protocol over the recruitment period [45]. Hwang et al. found equal survival (100%) but a lower root canal treatment rate in the rapid-prototyping group, a difference that persisted after excluding patients older than 40 years (50% versus 91%, *p* = 0.048) [38]. Kizek et al. reported success of 90% with replicas versus 71.4% without, an extra-alveolar time of 12 s versus 5.5 min, and ankylosis only in the conventional group, without formal statistical comparison [39].

Three studies compared two digital workflows. Chen et al. and Li et al. showed greater positional accuracy with a guide-plus-replica workflow than with a replica alone [22,44]; Li et al. also reported success of 100% versus 70% at 14 months, the difference arising from a nerve injury, periodontal pockets and fixation loosening in the replica-only arm, with no difference in fitting attempts or socket preparation time [44]. In the randomised comparison by Hou et al., a patient-specific replica required fewer re-preparations of the socket than a generic replica, but initial stability and healing at six months did not differ [23]. Keranmu et al. compared concentrated growth factor with no adjunct in replica-guided surgery and therefore provided no evidence on guidance itself; success was 100% versus 92.3% (not significant), and periapical healing was faster with the adjunct [36].

### 3.7. Risk of Bias

**Figure 3:**
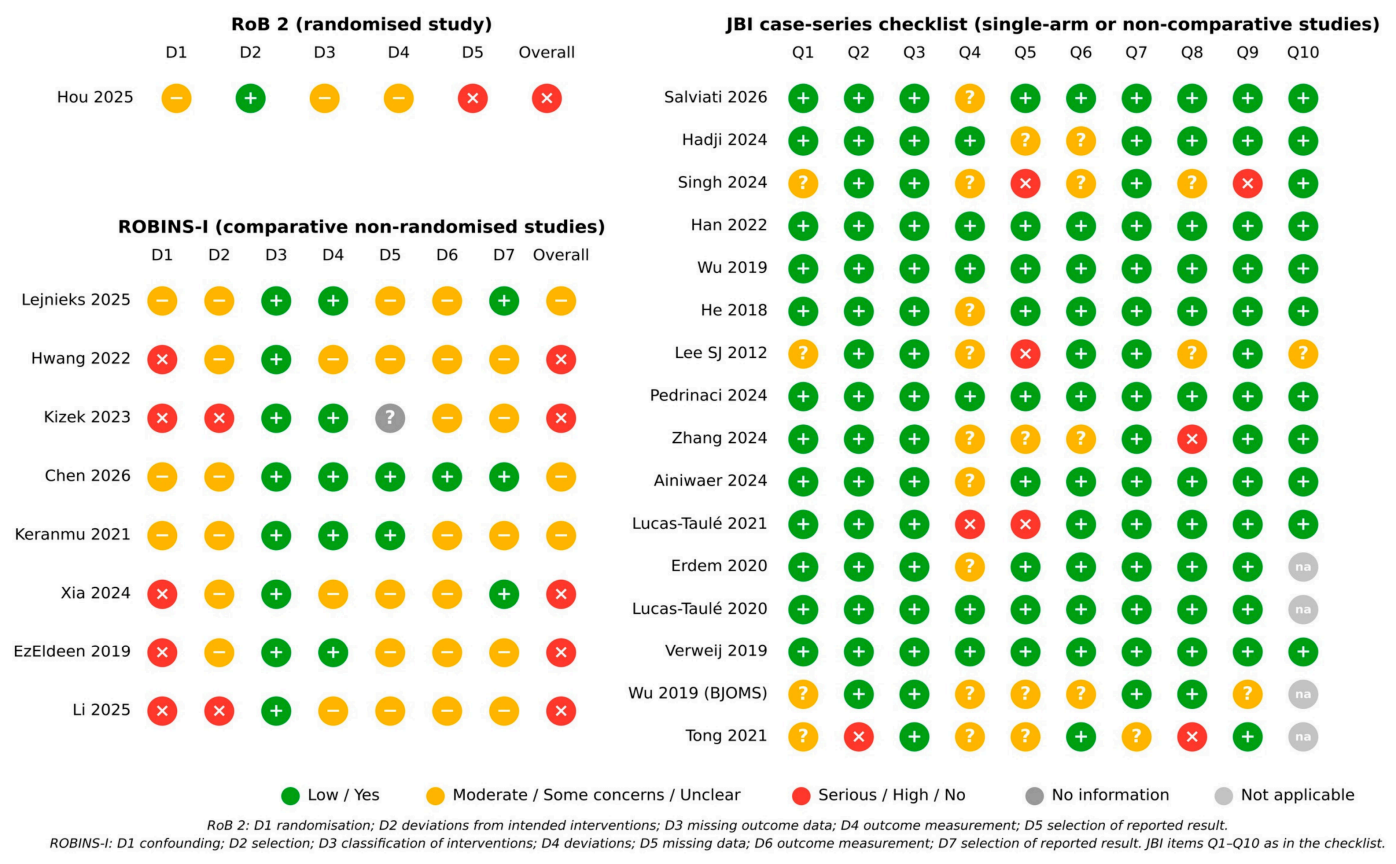
Risk of bias in the included studies. Top left, RoB 2 domain judgements for the randomised study [23] (D1 randomisation process; D2 deviations from intended interventions; D3 missing outcome data; D4 measurement of the outcome; D5 selection of the reported result). Bottom left, ROBINS-I domain judgements for the eight comparative non-randomised studies [22,36,38,39,41,44,45,53] (D1 confounding; D2 selection of participants; D3 classification of interventions; D4 deviations from intended interventions; D5 missing data; D6 measurement of outcomes; D7 selection of the reported result). Right, JBI case-series checklist responses for the sixteen single-arm or non-comparative studies [18,20,34,35,37,40,42,43,46,47,48,49,50,51,52,54] (Q1 to Q10 as in the checklist). Green circle with +, low risk or yes; yellow circle with −, moderate risk or some concerns; yellow circle with ?, unclear (JBI); red circle with ×, serious or high risk or no; dark grey circle with ?, no information; light grey circle with na, not applicable.

Risk-of-bias judgements are summarised in Figure 3 and Supplementary Table S13, with domain-level support in Supplementary Tables S14 and S15. The randomised study by Hou et al. was judged at high risk of bias with RoB 2, because allocation concealment was not described, no registration or protocol was available, the number of patients differed between the text and the tables, and operative time was claimed in the abstract but not reported [23]. Of the eight comparative studies assessed with ROBINS-I, three were judged at moderate overall risk of bias [22,36,41] and five at serious risk [38,39,44,45,53]. Confounding was the dominant concern: in the retrospective cohorts the groups differed in age, indication, operator or period of treatment, with adjustment in only one study [45]; in the Leuven cohort the conventional group was treated earlier [53]; and in the study by Li et al. the operator assigned participants and patients with intraoperative template fracture were excluded from the guided arm after allocation [44]. The blinding of outcome assessors was not reported in any comparative study. The registered trial by Lejnieks et al. was the only comparative study judged at low risk for selection of the reported result [41]. Of the sixteen single-arm studies appraised with the JBI checklist, seven met all or all but one item [20,34,35,37,43,46,51], four were judged at low to moderate risk because of unclear consecutive inclusion or incomplete tabulation of clinical outcomes [40,47,48,50], one at moderate risk because cases without virtual planning, with less than one year of follow-up or with intraoperative complications were excluded by design [49], two at moderate to high risk because of minimal numerical reporting in very small series [52,54], and two at high risk because of unexplained inconsistencies in their reported case numbers and, for one, the absence of any clinical follow-up [18,42].

### 3.8. Certainty of the Evidence

The certainty of the evidence for the comparison of digitally guided with conventional autotransplantation is summarised in Table 3. For survival, success, root resorption, ankylosis and the need for endodontic treatment, the evidence was rated very low. Although the number of comparative studies has grown and two now report follow-up beyond four years, every outcome is contributed by non-randomised studies of which most are at serious risk of bias from confounding; the direction of effect is consistent but its size varies from no difference to a large difference, and most studies report few events without confidence intervals. For extra-alveolar time, the evidence remained very low: four studies favoured guidance, but the effect ranged from a non-significant 9 s in the registered trial to more than five minutes in the retrospective cohorts, and the larger differences came from the studies at serious risk of bias. For total surgical time the evidence was rated low: the registered trial and the Hangzhou cohort reported differences of 13 and 15 min in the same direction, with a confidence interval or test in each. For positional accuracy of a guide-plus-replica workflow relative to a replica alone, the evidence was rated low: two small studies, one at moderate and one at serious risk of bias, reported consistent halving of the centre deviation with an objective CBCT-based outcome. For the randomised comparison of a patient-specific with a generic replica, the evidence was rated low for fitting re-preparations and for six-month healing, starting from high and downgraded for risk of bias, and for imprecision in a single unregistered study.

## 4. Discussion

### 4.1. Principal Findings

This review identified 25 clinical studies, reported in 33 publications, of digitally guided tooth autotransplantation. Three findings stand out. First, the technology in clinical use is narrower than the search anticipated: every included study relied on a CBCT- or CT-derived 3D-printed replica of the donor tooth, eleven added a printed or milled osteotomy guide, and none used dynamic navigation or robotic assistance, which so far appear only in proof-of-concept reports [21]. Second, the operative benefit is consistent. Extra-alveolar time was below one minute in eleven replica-guided reports, most donor teeth fitted at the first attempt, and where a conventional arm existed, surgery was shorter by about 13 to 15 min [45,55,57]. Third, the clinical benefit is plausible but unproven. Survival of 92% to 100% and success of 86% to 100% in the guided arms, now with follow-up extending to five years in three cohorts, are high, but similar figures are reported for conventional autotransplantation; the only registered controlled trial found no significant difference in survival, periodontal or radiographic outcomes once operative time and root development were taken into account [41], and the prospective paediatric cohort with the longest comparative follow-up found differences in survival and success of 8 points that did not reach significance [53]. The retrospective comparisons that favoured guidance, including the large Hangzhou cohort in which replica use was associated with a forty-fold difference in inflammatory resorption, were at serious risk of bias from confounding by period, operator and co-interventions [38,39,45].

### 4.2. Comparison with Published Syntheses

The clinical outcomes of the guided series can be set against the survival of autotransplanted teeth in general. Recent meta-analyses report short-term (1 to 4 years) survival of about 96% [8], overall survival of 87% to 96% and success of 84% to 92% irrespective of apex status [6,9,24,60], about 94% survival and 85% success for immature donors in children and adolescents [10], and survival above 95% at 5 and 10 years for open-apex donors [16]. The 92% to 100% survival in the guided series reviewed here is therefore at the upper end of, but not clearly above, this range. The long-term secondary reports from Seoul are a reminder that attrition continues beyond the follow-up of most guided series: survival of mature-tooth transplants performed with rapid-prototyped models fell from 88% at 3 years to 68% at 12 years [4]. One meta-analysis of mature third-molar autotransplantation reported lower pooled root resorption (2.1% versus 10.2%) and ankylosis (2.8% versus 6.5%) in studies using 3D techniques than in those without [5]; that comparison is between studies rather than within them, and is subject to the same confounding by period, operator and case selection that limits the retrospective comparisons in this review [38,39,45]. The only within-study comparison at trial level, by Lejnieks et al., remains neutral for clinical outcomes [41], while favouring guidance for operative time [55].

The heterogeneity of outcome definitions seen in this review reflects the field. Success was defined by Tsukiboshi criteria [35], Andreasen criteria [36], periodontal thresholds [49], the absence of pain, mobility and periapical pathology [39], or was not formally defined. The Autotransplantation Outcome Index proposed in the umbrella review by Tan et al. [16] and the calls for standardised reporting by Cremona et al. [14] and Baxmann et al. [7] apply with particular force to digitally guided studies, in which operative metrics such as extra-alveolar time, fitting attempts and positional deviation should also be recorded in a uniform way [26].

### 4.3. Reports Retrieved During Peer Review and Reports That Remain Unretrieved

At the time of the original search, 33 of the 54 reports sought could not be obtained through the library access, interlibrary loan and author requests available to the review team. During peer review, 30 of these were obtained through the publishers’ sites and the institutional library access of a collaborating researcher, and they were screened, extracted and appraised in the same way as the original set; the dated retrieval attempts for every report are documented in Supplementary Table S10. Thirteen of the 30 were included as primary studies and eight as secondary reports of cohorts already represented, and nine were excluded, including one retracted article. Their inclusion changed the review in three ways. It added the only randomised study [23] and the two comparative cohorts with follow-up beyond four years [45,53]; it widened the observed range of survival and success in guided arms by adding long-term cohorts in which late failures accumulated; and it corrected the operative findings attributed to the registered trial, whose primary operative report shows a reduction in surgical time but no significant reduction in extra-alveolar time or fitting attempts [55]. It did not change the direction of the findings or the certainty rating of any clinical outcome. Three reports remain unretrieved: two reports in a clinical orthodontic journal not available through any of the libraries accessed, which on their titles describe single cases and would not have met the sample-size threshold, and one report of an en bloc technique for which no digital guidance is described in the abstract; none is expected to have affected the synthesis.

### 4.4. Strengths and Limitations

The strengths of this review are a sensitive search of six databases and a trial registry without date restriction, prespecified eligibility criteria that excluded case reports, a design-matched risk-of-bias assessment using RoB 2, ROBINS-I and the JBI case-series checklist, explicit linking of multiple reports of the same cohort so that patients are counted once, and transparent reporting of internal inconsistencies within the included reports.

The principal limitations lie in the evidence rather than in its retrieval. Three of 54 reports sought remain unobtained (Section 4.3). Meta-analysis was not possible, so no pooled effect estimate is offered. Most included studies were single-centre, half enrolled fewer than 25 patients, and only three cohorts followed transplants for more than four years, so late complications such as replacement resorption, which is the main reason for tooth loss beyond four years [8], are under-represented. Several studies came from the same centres over overlapping periods; the primary report for each outcome was chosen as described, but residual double counting within the Seoul and Hangzhou cohorts cannot be excluded. The search was restricted to English-language records, and the review did not include the grey literature beyond a trial registry. The risk-of-bias judgements depend on what was reported; unreported allocation methods and blinding were treated as sources of uncertainty rather than assumed to be adequate, and the one randomised study could not be checked against a registration or protocol.

### 4.5. Implications for Practice and Research

The evidence supports two different claims to different degrees, and clinicians should keep them apart. The first is operative: a CBCT-derived 3D-printed replica, used to prepare and verify the recipient socket before the donor tooth is extracted, shortens extra-alveolar time to well under a minute in most hands, reduces fitting attempts, and shortens surgery by about a quarter of an hour; adding a printed osteotomy guide brings the achieved position to within about a millimetre of the plan. This claim rests on consistent findings across designs and is the practical basis for adopting the workflow, particularly for difficult recipient sites, multi-rooted donors and less experienced operators. The second claim is prognostic: that these operative gains translate into higher survival, fewer resorptive complications and less need for endodontic treatment. That claim is biologically plausible and is supported in direction by the retrospective comparisons, including one with five years of follow-up, but it is not confirmed by the one registered trial after adjustment or by the prospective paediatric cohort, and the certainty of the evidence is very low. Patients can therefore be told that digital guidance makes the procedure quicker and more predictable to perform, not that it has been shown to make the transplanted tooth last longer.

For researchers, the priorities are randomised or well-controlled prospective comparisons with allocation concealment, blinded outcome assessment, prospective registration and follow-up of at least two years; uniform reporting of extra-alveolar time, fitting attempts and positional deviation; adoption of a standard outcome index; and economic evaluation, since cost and access are recognised barriers to adoption [23,26].

## 5. Conclusions

Within the limits of the available evidence, digitally guided tooth autotransplantation, in practice a CBCT-based workflow with a 3D-printed donor tooth replica and, in some centres, a printed osteotomy guide, is associated with extra-alveolar times of under one minute, a first-attempt fit in most cases, positional deviations of about one to two millimetres from the virtual plan, and survival of 92% to 100% with success of 86% to 100% at six months to five years. These outcomes are promising and are consistent with the best results reported for conventional autotransplantation, but they do not demonstrate clinical superiority. The comparative evidence consists of one registered non-randomised trial and one prospective paediatric cohort that found no significant clinical difference, three retrospective cohorts at serious risk of bias that favoured guidance, and one randomised comparison of two replica types; the certainty of evidence is low for surgical time and positional accuracy and very low for every clinical outcome. Digital guidance can therefore be recommended as a means of making autotransplantation more efficient and more predictable to perform; whether it improves the prognosis of the transplanted tooth is a question that adequately powered, registered randomised trials with standardised outcomes and follow-up beyond two years have yet to answer.

## Figures and Tables

**Table 1:** Characteristics of the included studies (n = 25 studies; secondary reports in italics beneath the primary report).

Study (Country)	Design; Comparator	Patients/Teeth	Age	Donor Tooth; Apex	Follow-Up
Lee 2012 [18] (South Korea)	Retrospective series; none	182/251 cases reported *	Mean 36 y (13–67)	Third molars; mature	Not reported
*Kim 2005 [58]; Lee 2001 [17]; Lee 2004 [59]; Jang 2016 [4] (same centre, 2001–2010)*	*Series (182); series (22); series (60); prognostic cohort (105 teeth)*	*182/182; 22/22; 60; 96/105*	*27–58 y (Lee 2001 [17]); otherwise NR*	*Mostly mandibular molars; mature*	*2–60 mo; ≥1 y; none; up to 12 y*
He 2018 [34] (China)	Prospective series; none	8/8	Mean 26.9 y (23–32)	Third molars; mature	Mean 2.0 y (1–4)
Wu 2019 [35] (China)	Retrospective series; none	10/10	Mean 31.6 y (19–42)	Impacted third molars; mature	2 y
Wu 2019 [52] (China)	Case series; none	5/5	21–41 y	Not tabulated (third molar in the illustrated case); mature †	6 mo
EzEldeen 2019 [53] (Belgium)	Prospective comparative cohort; conventional (n = 50 teeth)	88 (44 guided)/100 (50 guided)	Mean 10.7 y guided; 10.6 y conventional	Premolars; immature	Mean 4.5 y (1.1–10.4)
*Shahbazian 2013 [57] (same centre)*	*Case–control; historical conventional (n = 24 teeth)*	*40 (20 guided)/48 (24 guided)*	*Mean 11 y guided; 12 y control*	*Premolars (22) and molars (2) per arm; immature*	*1 y*
Verweij 2020 [51] (Netherlands)	Prospective multicentre series; none	79/100	Mean 13.0–16.6 y by tooth type	Premolars (73), molars (24), canines (2), supernumerary (1); mostly 50–90% root length	None (intraoperative only)
*Verweij 2016 [19] (same centre)*	*Prospective proof of concept; none*	*5/5*	*NR*	*Maxillary premolars; developing*	*None (intraoperative only)*
Lucas-Taulé 2020 [20] (Spain)	Prospective consecutive series; none	10/10	Mean 16.6 y (9–37)	Premolars (6, open apex), third molars (4, closed)	Mean 13.1 mo (6–27)
Erdem 2021 [50] (Turkey)	Retrospective series; none	10/12	15–21 y	Impacted third molars; immature	Mean 20.4 mo (≥18)
Keranmu 2021 [36] (not stated ‡)	Two-arm comparison; CGF vs. no CGF, replica in both arms	52 (51 evaluated)/52	Mean 32.6 y (21–46)	Third molars; mature	18–36 mo (mean 26)
Lucas-Taulé 2021 [49] (Spain)	Retrospective cohort; none	36/36	Mean 30.2 y (14–61)	Third molars; 12 open, 24 closed apex	Mean 29.4 mo
Tong 2021 [54] (Australia)	Case series; none	12/15	10–24 y	Premolars and third molars; 14 immature, 1 mature	6 mo (imaging to 12 mo)
Han 2022 [37] (China)	Retrospective cohort; post hoc seated vs. not-seated	41/41	Mean 28.7 y (18–48)	Third molars; mostly mature	1 y (periodontal healing at 6 mo)
Hwang 2022 [38] (Taiwan)	Retrospective cohort; conventional (n = 13)	21 (8 guided)/21	21.6 y guided; 28.8 y conventional	Mostly third molars; mature	≥12 mo (guided); >6 mo (all)
Kizek 2023 [39] (not stated ‡)	Retrospective cohort; no replica (n = 14)	24 (10 guided)/not reported	16.2 y guided; 21.9 y conventional	Mainly impacted maxillary canines; not reported	12–36 mo (mean 14)
Ainiwaer 2024 [48] (China)	Retrospective cohort; created vs. fresh socket (replica in both arms)	96/96	Mean 29.9 and 31.7 y (22–52)	Third molars; mature	Mean 47.6 mo (18–78)
Hadji 2024 [40] (France)	Retrospective consecutive series; none	24 (20 evaluated)/26 (22 evaluated)	Median 15 y	Mostly second premolars; mixed (9 immature)	Median 23–25.5 mo
Pedrinaci 2024 [46] (Spain)	Prospective series; none (enamel matrix derivative in all)	12/13	Mean 35.6 y (23–58)	Third molars; mature	2 y
Singh 2024 [42] (not stated ‡)	Prospective multicentre series; none	152/200 (160 in one passage) *	13–16.5 y by tooth type	Premolars (146), molars (48), canines (4); mostly open apex	None (intraoperative only)
Xia 2025 [45] (China)	Retrospective cohort; no replica (n = 68 teeth)	160/168 (100 guided)	Mean 29.6 y	Third molars; mature	Mean 5.2 y
*Xia 2020 [56] (same centre)*	*Series; none*	*27/28*	*Mean 27.6 y (20–34)*	*Third molars; mature*	*2 y*
Zhang 2024 [47] (China)	Retrospective accuracy study; none	48/50	Adults (not stated)	Mixed (29 single-root, 21 multi-root); 48 mature	Immediate CBCT (clinical to 12 mo, not tabulated)
Lejnieks 2025 [41] (Latvia)	Non-randomised controlled trial; conventional (n = 22)	46 (24 guided)/46	Median 16 y guided; 17.5 y conventional	Unerupted third molars; immature	12 mo
*Lejnieks 2024 [55] (same trial, operative outcomes)*	*As above*	*46 (24 guided)/46*	*13–22 y*	*As above*	*Intraoperative; 3–4 wk*
Hou 2025 [23] (China)	Randomised comparison; patient-specific replica (n = 56) vs. universal replica (n = 60)	117 stated/116 in tables *	Mean 32.3 y (18–60)	Third molars; mature	6 mo
Li 2025 [44] (China)	Non-randomised comparison (titled randomised §); replica only (n = 15)	29/30 (15 guided)	Mean 28.2 y (18–42)	Third molars; mature	Mean 14.9 mo guided; 14.2 mo control
Chen 2026 [22] (China)	Prospective non-randomised comparison; replica-only freehand (n = 12)	24 (12 guided)/24	Not reported (16–60 y eligible)	Third molars; not reported	None (immediate CBCT)
Salviati 2026 [43] (Italy)	Prospective cohort; none	10/10	Mean 27.2 y (18–32)	Erupted third molars; mature	18 mo

CBCT, cone beam computed tomography; CGF, concentrated growth factor; NR, not reported. * Internally inconsistent counts as reported in the paper. † Donor type not tabulated in the report; inferred from the surgical narrative. ‡ Country of the clinical setting not stated in the report; author affiliations indicate Slovakia (Kizek et al.), China (Keranmu et al.) and India (Singh et al.). § The appendix states that no random allocation sequence was generated and that the operator assigned participants.

**Table 2:** Clinical and radiographic outcomes of digitally guided autotransplantation.

Study	Survival	Success (Criteria)	Root Resorption	Ankylosis	Endodontic Treatment
Lee 2012 [18]	NR	NR	4/251 (1.6%); follow-up NR	NR	RCT before extraction in most (168 intra-oral, 9 extra-oral)
*Kim 2005 [58]; Jang 2016 [4]*	*Failure 9/182 (4.5%) over 2–60 mo; cumulative survival 88.1% at 3 y and 68.2% at 12 y*	*Complete healing 112/143 at first follow-up; NR*	*4/182 (2.4%); IRR 12.1% at 3 y*	*18/182 (10.7%); 27.8% at 2 y (plateau)*	*RCT before extraction where accessible*
He 2018 [34]	100% at 1 y (8/8)	87.5% (7/8)	None	1/8	All teeth
Wu 2019 [35]	All in function at 2 y	100% at 2 y (Tsukiboshi)	None at 2 y	None	All teeth (mature donors)
Wu 2019 [52]	100% at 6 mo (5/5)	5/5 (no resorption or radiolucency; one tooth grade 1 mobility)	None	None	All teeth, 2 wk after surgery
EzEldeen 2019 [53]	92% guided vs. 84% conventional, mean 4.5 y	86% vs. 78% (not significant)	Reported within failure categories	NR separately	Not applicable (immature donors)
*Shahbazian 2013 [57]*	*24/24 vs. 22/24 at 1 y*	*22/24 vs. 18/24*	*Infection-related 0 vs. 2*	*Replacement resorption 1 vs. 4*	*Not applicable*
Verweij 2020 [51]; *Verweij 2016 [19]*	NR (operative study)	NR	NR	NR	NR
Lucas-Taulé 2020 [20]	100% at mean 13 mo (10/10)	100% (no pathologic mobility, inflammation, radiolucency or resorption)	1 inflammatory resorption, arrested after RCT	None	4/4 closed-apex teeth within 1 mo; open-apex teeth vital
Erdem 2021 [50]	100% at mean 20 mo (12/12)	100% (in function, no clinical or radiographic pathology)	None	None	None (all vital)
Keranmu 2021 [36]	NR separately	100% CGF vs. 92.3% control, *p* > 0.05 (Andreasen)	1 case (control) at 2 y	None (CGF); NR control	NR
Lucas-Taulé 2021 [49]	97.2% (35/36), mean 29 mo	91.7% (33/36) (PPD ≤ 4 mm, CAL ≤ 5 mm, no progressive resorption)	Replacement 1; inflammatory 0	1	All 24 closed-apex teeth within 1 mo; none of 12 open-apex
Tong 2021 [54]	100% at 6 mo (15/15)	All vital and stable at 6 mo (no formal criteria)	None reported	None reported	None (all vital)
Han 2022 [37]	No difference between groups; 1 extracted	Periodontal healing 38/41 at 6 mo	1 case	NR	RCT in 29 at 2–4 wk; 1 delayed
Hwang 2022 [38]	100% both groups	NR	NR	NR	59% (50% in text) guided vs. 92.3% conventional
Kizek 2023 [39]	NR separately	90% guided vs. 71.4% conventional (no pain, mobility or periapical pathology)	NR	0/10 guided vs. 3/14 conventional	3 vs. 4 patients
Ainiwaer 2024 [48]	95.8% (92/96), mean 47.6 mo	95.8% (failure defined as tooth loss)	Progressive resorption 2/96	NR separately	90/96 within 4 wk
Hadji 2024 [40]	95.5% (21/22), median 23 mo	90.5% (19/21), median 25.5 mo	None	None	2 immature teeth; 13 mature teeth by protocol
Pedrinaci 2024 [46]	100% at 2 y (13/13)	91.2% as reported (12/13 = 92.3%) *	No progressive resorption	None	All teeth (mature donors)
Singh 2024 [42]	NR	NR	NR	NR	NR
Xia 2025 [45]; *Xia 2020 [56]*	99% replica vs. 79% no replica (99/100 vs. 54/68), mean 5.2 y; *28/28 at 2 y*	98% vs. 41% (98/100 vs. 28/68); *28/28*	Inflammatory 1% vs. 41% (1/100 vs. 28/68), *p* < 0.001; *none*	Within success criteria	All teeth; timing ≤ 4 wk associated with success
Zhang 2024 [47]	“All cases successful” at 12 mo (not tabulated)	NR	NR	NR	Closed-apex teeth at 2–4 wk
Lejnieks 2025 [41]; *Lejnieks 2024 [55]*	100% at 1 y both groups	NR as a rate; mobility 0% vs. 13.6%	13% guided vs. 36% conventional, *p* = 0.057	NR	NR
Hou 2025 [23]	Failures at 6 mo: 1/25 and 2/31 (patient-specific) vs. 0/25 and 3/35 (universal), maxillary and mandibular sites *	Success 25/25 and 29/31 vs. 25/25 and 32/35, *p* = 0.33 and 0.75 *	Within failure criteria	Within failure criteria	All teeth (completed RCT is a success criterion)
Li 2025 [44]	100% both arms, mean 14 mo	100% guided vs. 70% replica only (no complications)	PDL continuity, no difference	NR	By protocol
Chen 2026 [22]	NR (accuracy study)	NR	NR	NR	NR
Salviati 2026 [43]	All stable at 18 mo	NR as a rate	None at 18 mo	None reported	2/10

CAL, clinical attachment level; CGF, concentrated growth factor; IRR, inflammatory root resorption; NR, not reported; PDL, periodontal ligament; PPD, probing pocket depth; RCT, root canal treatment. Values are for the digitally guided group unless a comparison is shown. Secondary reports in italics. * Internally inconsistent values as reported in the paper.

**Table 3:** Summary of findings and GRADE certainty of evidence for digitally guided versus conventional autotransplantation, and for comparisons between digital workflows.

Outcome	Studies (Participants)	Risk of Bias	Inconsistency	Indirectness	Imprecision	Effect (as Reported)	Certainty
**Guided vs. conventional**							
Survival, 12 months to 5 years	4 NRS (315) [38,41,45,53]	Serious (three studies)	Not serious (direction consistent; size 0–20 points)	Not serious	Serious (few events; CI only in one study)	100% vs. 100% at 1 y (two studies); 92% vs. 84% at 4.5 y (NS); 99% vs. 79% at 5.2 y	Very low
Success, 12 months to 5 years	3 NRS (272) [39,45,53]	Serious	Not serious	Not serious	Very serious	90% vs. 71%; 86% vs. 78% (NS); 98% vs. 41%	Very low
Root resorption	2 NRS (206) [41,45]	Serious (one study)	Serious (near-null vs. large)	Not serious	Serious	13% vs. 36%, *p* = 0.057; 1% vs. 41%, *p* < 0.001	Very low
Ankylosis	1 NRS (24) [39]	Serious	Not applicable	Not serious	Very serious	0/10 vs. 3/14	Very low
Need for endodontic treatment	1 NRS (21) [38]	Serious	Not applicable	Not serious	Very serious	50–59% vs. 91–92%, *p* = 0.048	Very low
Extra-alveolar time	4 NRS (318) [39,41,45,53]	Serious (three studies)	Serious (−9 s, NS, to > 5 min)	Not serious	Serious	Shorter with guidance in all; significant in two	Very low
Total surgical time	3 NRS (294) [41,45,53]	Serious (two studies)	Not serious (−13 to −15 min)	Not serious	Not serious	−13.4 min (95% CI −24.5 to −2.3); 45.6 vs. 60.5 min, *p* < 0.001	Low
**Comparisons between digital workflows**							
Positional accuracy: guide + replica vs. replica alone	2 NRS (53) [22,44]	Serious (one study)	Not serious	Serious (different comparator)	Serious; large consistent effect	Centre deviation 1.00 vs. 2.00 mm; 0.79 vs. 1.79 mm	Low
Socket re-preparation: patient-specific vs. generic replica	1 RCT (116) [23]	Serious (high RoB 2)	Not applicable	Serious (generic replica comparator)	Serious	Fewer re-preparations with patient-specific replica, *p* = 0.025 and 0.042	Low
Six-month healing: patient-specific vs. generic replica	1 RCT (116) [23]	Serious	Not applicable	Serious	Serious (few events)	No difference, *p* = 0.33 and 0.75	Low

CI, confidence interval; GRADE, Grading of Recommendations Assessment, Development and Evaluation; NRS, non-randomised study; NS, not significant; RCT, randomised controlled trial. Certainty starts at low for non-randomised studies and at high for the randomised study. Single-arm studies did not contribute to comparative certainty ratings.

## Data Availability

All data generated or analysed during this review are included in this article and its Supplementary Materials: the search strategies, screening decisions and retrieval log (Files S1–S3), the data extraction (Files S1 and S4) and the risk-of-bias assessments (Supplementary Tables S13–S15). The review protocol is registered in PROSPERO CRD420261434791.

## Supplementary Materials

The following supporting information can be downloaded at: https://www.mdpi.com/article/10.3390/jcm15218601/s1. File S1 (Supplementary_Materials.docx): Table S1: MEDLINE (PubMed) search strategy; Table S2: MEDLINE (Ovid) search strategy; Table S3: Embase (Ovid) search strategy; Table S4: Scopus search strategy; Table S5: Web of Science Core Collection search strategy; Table S6: Cochrane Library (CENTRAL) search strategy; Table S7: ClinicalTrials.gov registry search strategy; Table S8: Google Scholar supplementary search strategy; Table S9: Reports excluded at full-text assessment, with reasons (n = 18); Table S10: Retrieval log for the 33 reports that could not be obtained at the time of the original search (August 2026), with the date, route and outcome of each attempt; Table S11: Digital workflows used in the included studies; Table S12: Operative and accuracy outcomes; Table S13: Summary of risk-of-bias judgements for the 25 included studies; Table S14: RoB 2 domain-level judgements for the randomised study and ROBINS-I domain-level judgements for the eight comparative non-randomised studies, with supporting statements; Table S15: JBI case-series checklist item-level judgements and supporting statements for the sixteen single-arm or non-comparative studies; Table S16: Data extraction for the 25 included studies and their secondary reports; Table S17: PRISMA 2020 checklist; Table S18: PRISMA-S checklist for literature searches. File S2 (Screening_Strategy.docx): Search sources, search strategy and screening process. File S3 (Included_and_Excluded_Studies.xlsx): Included reports, reports excluded at full-text assessment and the retrieval log, in spreadsheet form. File S4 (Data_Extraction_Included_Studies.xlsx): Data extraction and outcome data for the included studies, in spreadsheet form. References [61,62,63,64,65,66,67,68,69,70,71,72,73,74,75,76,77,78,79] are cited in the Supplementary Materials.
