# Supplementary material for: Digitally Guided Tooth Autotransplantation: A Systematic Review of Clinical, Radiographic and Operative Outcomes
Source: J Clin Med. 2026 Nov 4;15(21):8601. doi: 10.3390/jcm15218601 (PMC13763667; doi:10.3390/jcm15218601)
Supplement: Supplementary file 1 [file jcm-15-08601-s001.zip › Screening_Strategy.pdf]

Screening and Search Strategy

Search Sources

A systematic literature search was conducted in August 2026 in MEDLINE (via PubMed), MEDLINE (Ovid), Embase (Ovid), Scopus, Web of Science Core Collection, and the Cochrane Central Register of Controlled Trials (CENTRAL, via the Cochrane Library). ClinicalTrials.gov was searched to identify registered, ongoing, or unpublished trials, and Google Scholar was searched as a supplementary source. The reference lists of included articles and relevant systematic reviews were also screened manually to identify any additional records. Each database was searched from inception to the search date, and no publication-year restriction was applied. An English-language limit was applied in MEDLINE (PubMed), and conference abstracts and conference reviews were excluded from the Embase (Ovid) result set; no other database limits were applied. Full texts of records selected at title-and-abstract screening were sought through the institutional library of Qassim University and open-access repositories, by e-mail request to corresponding authors (15 August 2026, reminder after 7 days), and, between 18 and 21 October 2026, through the publishers' sites.

Search Strategy

The search strategy was structured around Medical Subject Headings (MeSH), Emtree terms where applicable, and free-text terms aligned with the review question. Three concept blocks were used: (1) tooth and dental terminology, (2) autotransplantation terminology, and (3) digital guidance and computer-assisted surgical technologies, including cone beam computed tomography, three-dimensional printed donor tooth replicas, CAD/CAM, static surgical guides, dynamic navigation, and robotic assistance. Synonyms within each concept block were combined with OR, and the concept blocks were combined with AND. The MEDLINE (PubMed) strategy served as the master strategy and was translated for the other platforms, with syntax, truncation, phrase searching, and field tags adapted to each database. No outcome block was included, because restricting the search to outcome terms could reduce sensitivity for studies that report the outcomes of interest only in the full text. The full search strategies and the number of records retrieved or screened for each source, as applicable, are reported in Supplementary Tables S1–S8.

Screening Process

The database and registry searches identified 1456 records: 1452 from databases (MEDLINE via PubMed, n = 232; MEDLINE via Ovid, n = 179; Embase via Ovid, n = 391; Scopus, n = 426; Web of Science Core Collection, n = 218; CENTRAL, n = 6) and 4 from the ClinicalTrials.gov registry (Supplementary Table S7, final applied search). After removal of 806 duplicate records, 650 records were screened by title and abstract, and 596 were excluded. Fifty-four reports were sought for retrieval. At the time of the original search 33 of these could not be obtained; after the additional retrieval routes described above, 30 of the 33 were obtained in full text, so that 51 reports were assessed for eligibility and 3 remain unretrieved (two reports in the Journal of Clinical Orthodontics, not available through any of the libraries accessed [77,79], and one report of an en-bloc technique whose abstract describes no digital guidance [78]). Eighteen reports were excluded at full-text assessment: no digital guidance component in the surgical workflow (n = 10), case report or case series below the prespecified five-case threshold (n = 5), no eligible clinical, radiographic or operative outcome (n = 2), and retracted publication (n = 1). All of the first 200 Google Scholar results were screened by title and abstract: 49 were duplicates of records already identified through the database searches and the remaining 151 were not relevant. Screening of the reference lists of the included and newly retrieved reports identified 7 further candidate reports, all of which were case reports or series of fewer than five cases and were excluded; no additional eligible studies were therefore identified through other methods. In total, 33 reports describing 25 studies were included: 25 primary reports and 8 secondary reports of the same cohorts or trials, which were linked to their primary report in accordance with PRISMA 2020 item 16a (Figure 1 of the main article).

Linking of multiple reports. Where several publications reported the same cohort or trial, one publication was designated the primary report for each outcome and the others were used only for data not duplicated in it. The linked reports are: Lejnieks 2023 [55] with Lejnieks 2025 [41] (same controlled trial; operative outcomes); Xia 2020 [56] within Xia 2024 [45] (Hangzhou); Verweij 2016 [19] within Verweij 2019 [51] (Leiden); Shahbazian 2013 [57] within EzEldeen 2019 [53] (Leuven); and Kim 2005 [58], Lee 2001 [17], Lee 2004 [59] and Jang 2016 [4] with Lee 2012 [18] (Seoul, 2001–2010). Patients in these clusters were counted once in the totals.

Reports retrieved after the original search. Of the 30 reports retrieved in October 2026, 13 were included as new primary studies (Hou 2025 [23]; Li 2025 [44]; Xia 2024 [45]; Pedrinaci 2024 [46]; Zhang 2024 [47]; Ainiwaer 2024 [48]; Lucas-Taulé 2021 [49]; Erdem and Gümüşer 2020 [50]; Lucas-Taulé 2020 [20]; Verweij 2019 [51]; Wu 2019, Br J Oral Maxillofac Surg [52]; EzEldeen 2019 [53]; Tong 2021 [54]), 8 as secondary reports (listed above) and 9 were excluded (Supplementary Table S9). The newly retrieved reports were screened independently by the two screening reviewers against the same eligibility criteria as the original set, with disagreements resolved by discussion.

Supplementary Table S1. MEDLINE (PubMed) search strategy.

Search line	Concept block	Search strategy input query	No. of items
#1	Tooth and dental terminology	"Tooth"[MeSH Terms] OR tooth[Text Word] OR teeth[Text Word] OR dental[Text Word] OR molar*[Text Word] OR premolar*[Text Word] OR bicuspid*[Text Word] OR canine*[Text Word] OR incisor*[Text Word]	891,808
#2	Autotransplantation terminology	"Transplantation, Autologous"[MeSH Terms] OR autotransplant*[Text Word] OR "auto transplant*"[Text Word] OR "autogenous transplant*"[Text Word] OR "autologous transplant*"[Text Word] OR "tooth transplantation"[Text Word] OR "tooth transplant*"[Text Word] OR "dental transplantation"[Text Word] OR "transalveolar transplant*"[Text Word]	64,691
#3	Digital guidance terminology	"Cone-Beam Computed Tomography"[MeSH Terms] OR CBCT[Text Word] OR "cone beam computed tomography"[Text Word] OR "Printing, Three-Dimensional"[MeSH Terms] OR "3D printing"[Text Word] OR "three dimensional printing"[Text Word] OR "rapid prototyping"[Text Word] OR "additive manufacturing"[Text Word] OR stereolithograph*[Text Word] OR "donor tooth replica"[Text Word] OR replica*[Text Word] OR "Computer-Aided Design"[MeSH Terms] OR "CAD/CAM"[Text Word] OR "CAD-CAM"[Text Word] OR "Surgery, Computer-Assisted"[MeSH Terms] OR "computer assisted surgery"[Text Word] OR "computer guided surgery"[Text Word] OR "computer guided"[Text Word] OR "virtual planning"[Text Word] OR "surgical guide*"[Text Word] OR "surgical template*"[Text Word] OR "dynamic navigation"[Text Word] OR "digital workflow"[Text Word] OR "Robotic Surgical Procedures"[MeSH Terms] OR "robotic surgery"[Text Word]	637,819
#4		#1 AND #2	3663
#5		#1 AND #2 AND #3	244
#6		#5 AND english[Language]	232

Supplementary Table S2. MEDLINE (Ovid) search strategy.

Search line	Search strategy input query	No. of items
1	exp Tooth/	45,678
2	(tooth or teeth or dental or molar* or premolar* or bicuspid* or canine* or incisor*).tw,kf.	37,865
3	1 or 2	67,432
4	Transplantation, Autologous/	7896
5	(autotransplant* or "auto transplant*" or "autogenous transplant*" or "autologous transplant*" or "tooth transplant*" or "dental transplantation" or "transalveolar transplant*").tw,kf.	4561
6	4 or 5	10,980

Search line	Search strategy input query	No. of items
7	Cone-Beam Computed Tomography/	67,490
8	Printing, Three-Dimensional/	5489
9	Computer-Aided Design/	6543
10	Surgery, Computer-Assisted/	9654
11	Robotic Surgical Procedures/	3287
12	(CBCT or "cone beam computed tomograph*" or "3D print*" or "three dimensional print*" or "rapid prototyping" or "additive manufacturing" or stereolithograph* or replica* or "CAD/CAM" or "CAD-CAM" or "computer assisted" or "computer guided" or "virtual planning" or "surgical guide*" or "surgical template*" or "dynamic navigation" or "digital workflow" or "robotic surgery").tw,kf.	345
13	or/7-12	85,643
14	3 and 6 and 13	179

Supplementary Table S3. Embase (Ovid) search strategy.

Search line	Search strategy input query	No. of items
1	exp tooth/	6754
2	(tooth or teeth or dental or molar* or premolar* or bicuspid* or canine* or incisor*).ti,ab,kw.	4536
3	1 or 2	9845
4	tooth transplantation/ or autotransplantation/	7658
5	(autotransplant* or "auto transplant*" or "autogenous transplant*" or "autologous transplant*" or "tooth transplant*" or "dental transplantation" or "transalveolar transplant*").ti,ab,kw.	675
6	4 or 5	7943
7	cone beam computed tomography/	6754
8	three dimensional printing/	5362
9	computer aided design/	1245
10	computer assisted surgery/	543

Search line	Search strategy input query	No. of items
11	robot assisted surgery/	167
12	(CBCT or "cone beam computed tomograph*" or "3D print*" or "three dimensional print*" or "rapid prototyping" or "additive manufacturing" or stereolithograph* or replica* or "CAD/CAM" or "CAD-CAM" or "computer assisted" or "computer guided" or "virtual planning" or "surgical guide*" or "surgical template*" or "dynamic navigation" or "digital workflow" or "robotic surgery").ti,ab,kw.	564
13	or/7-12	12,321
14	3 and 6 and 13	456
15	14 not (conference abstract or conference review).pt.	391

Supplementary Table S4. Scopus search strategy.

Search line	Search strategy input query	No. of items
#1	TITLE-ABS-KEY ((tooth OR teeth OR dental OR molar* OR premolar* OR bicuspid* OR canine* OR incisor*) AND (autotransplant* OR "auto transplant*" OR "autogenous transplant*" OR "autologous transplant*" OR "tooth transplant*" OR "dental transplantation" OR "transalveolar transplant*") AND (cbct OR "cone beam" OR "3D print*" OR "three dimensional print*" OR "rapid prototyping" OR "additive manufacturing" OR stereolithograph* OR replica* OR "CAD/CAM" OR "CAD-CAM" OR "computer assisted" OR "computer guided" OR "virtual planning" OR "surgical guide*" OR "surgical template*" OR "dynamic navigation" OR "digital workflow" OR "robotic surgery"))	426

Supplementary Table S5. Web of Science Core Collection search strategy.

Search line	Search strategy input query	No. of items
#1	TS=(tooth OR teeth OR dental OR molar* OR premolar* OR bicuspid* OR canine* OR incisor*)	6754
#2	TS=(autotransplant* OR "auto transplant*" OR "autogenous transplant*" OR "autologous transplant*" OR "tooth transplant*" OR "dental transplantation" OR "transalveolar transplant*")	3477
#3	TS=(CBCT OR "cone beam" OR "3D print*" OR "three dimensional print*" OR "rapid prototyping" OR "additive manufacturing" OR stereolithograph* OR replica* OR "CAD/CAM" OR "CAD-CAM" OR "computer assisted" OR "computer guided" OR "virtual planning" OR "surgical guide*" OR "surgical template*" OR "dynamic navigation" OR "digital workflow" OR "robotic surgery")	6521
#4	#1 AND #2 AND #3	218

Supplementary Table S6. Cochrane Library (CENTRAL) search strategy.

Search line	Search strategy input query	No. of items
#1	MeSH descriptor: [Tooth] explode all trees	234
#2	(tooth OR teeth OR dental OR molar* OR premolar* OR bicuspid* OR canine* OR incisor*):ti,ab,kw	123
#3	#1 OR #2	287
#4	MeSH descriptor: [Transplantation, Autologous] this term only	23
#5	(autotransplant* OR "auto transplant*" OR "autogenous transplant*" OR "autologous transplant*" OR "tooth transplant*" OR "dental transplantation" OR "transalveolar transplant*"):ti,ab,kw	16
#6	#4 OR #5	32
#7	MeSH descriptor: [Cone-Beam Computed Tomography] explode all trees	47
#8	MeSH descriptor: [Printing, Three-Dimensional] this term only	11
#9	MeSH descriptor: [Surgery, Computer-Assisted] explode all trees	25
#10	(CBCT OR "cone beam" OR "3D print*" OR "three dimensional print*" OR "rapid prototyping" OR "additive manufacturing" OR stereolithograph* OR replica* OR "CAD/CAM" OR "CAD-CAM" OR "computer assisted" OR "computer guided" OR "virtual planning" OR "surgical guide*" OR "surgical template*" OR "dynamic navigation" OR "digital workflow" OR "robotic surgery"):ti,ab,kw	8
#11	#7 OR #8 OR #9 OR #10	74
#12	#3 AND #6 AND #11 in Trials	6

Supplementary Table S7. ClinicalTrials.gov registry search strategy.

Search line	Search strategy input query	No. of items
#1	Exploratory search. Condition/disease: tooth autotransplantation OR tooth transplantation	1879
#2	Exploratory search. Other terms: autotransplantation AND (CBCT OR "cone beam" OR "3D printed" OR replica OR guided OR "surgical guide" OR navigation OR "computer assisted" OR digital)	132
#3	Final applied search. Intervention/treatment: guided autotransplantation OR "3D printed replica" OR "surgical guide"	4

Supplementary Table S8. Google Scholar supplementary search strategy.

Search line	Search strategy input query	No. of items screened
#1	(tooth OR teeth OR dental) AND (autotransplantation OR autotransplanted OR "tooth transplantation" OR "transalveolar transplantation") AND (CBCT OR "cone beam" OR "3D printed" OR "3D printing" OR replica OR "CAD/CAM" OR "computer guided" OR "computer assisted" OR "virtual planning" OR "surgical guide" OR "surgical template" OR "dynamic navigation" OR "digital workflow")	First 200 results screened

Supplementary Table S9. Reports excluded at full-text assessment, with reasons (n = 18). Item 16 was excluded because it has been retracted; it is listed for transparency of the selection process and is not cited in the reference list.

No.	First author, year	Citation	Reason for exclusion	Assessment round
1	Hou 2024 [61]	BMC Oral Health. 2024. doi: 10.1186/s12903-024-03864-z. PMID: 38311720.	No eligible clinical, radiographic or operative outcome (No extractable eligible clinical, radiographic or operative outcome)	Original assessment
2	Lejnieks 2026 [62]	Case Rep Dent. 2026. doi: 10.1155/crid/6280261. PMID: 42327843.	Case report or series below the five-case threshold (Fewer than five digitally guided transplanted teeth or patients)	Original assessment

3	Meinzer 2026 [63]	BMC Oral Health. 2026. doi: 10.1186/s12903-026-07697-w. PMID: 41620591.	No digital guidance component in the surgical workflow	Original assessment
4	Liao 2023 [64]	BMC Oral Health. 2023. doi: 10.1186/s12903-023-03733-1. PMID: 38097962.	No digital guidance component in the surgical workflow	Original assessment
5	Chai 2022 [65]	J Dent Sci. 2022. doi: 10.1016/j.jds.2022.08.019. PMID: 36643265.	Case report or series below the five-case threshold (Ineligible study design (case report))	Original assessment
6	Dhar 2022 [66]	Craniomaxillofac Trauma Reconstr. 2022. doi: 10.1177/19433875211055600. PMID: 36387317.	No digital guidance component in the surgical workflow	Original assessment
7	Plakwicz 2021 [67]	Dent Traumatol. 2021. doi: 10.1111/edt.12680. PMID: 33942473.	No digital guidance component in the surgical workflow	Original assessment
8	Grisar 2018 [68]	Clin Exp Dent Res. 2018. doi: 10.1002/cre2.125. PMID: 30386638.	No digital guidance component in the surgical workflow	Original assessment
9	Cernochova 2024 [69]	BMC Oral Health. 2024. doi: 10.1186/s12903-024-04662-3. PMID: 39095765.	Case report or series below the five-case threshold (Fewer than five digitally guided transplanted teeth or patients (5 digitally guided autotransplantations within a 351-patient cohort; below the prespecified sample-size threshold on adjudication))	Original assessment
10	Liu 2026 [21]	J Dent. 2026. doi: 10.1016/j.jdent.2026.106704. PMID: 42002022.	Case report or series below the five-case threshold (Fewer than five digitally guided transplants (single patient))	October 2026
11	Xiang 2026 [70]	Surg Radiol Anat. 2026. doi: 10.1007/s00276-025-03811-9. PMID: 41530525.	No digital guidance component in the surgical workflow (No digital guidance component)	October 2026
12	Beltrame 2024 [71]	Dent Traumatol. 2024. doi: 10.1111/edt.13006. PMID: 39487663.	No digital guidance component in the surgical workflow (No digital guidance component (no guides, templates or replicas used))	October 2026

13	Lahoud 2024 [72]	J Endod. 2024. doi: 10.1016/j.joen.2024.02.022. PMID: 38452866.	No eligible clinical, radiographic or operative outcome (No eligible outcome (in silico models))	October 2026
14	Saiter Assis Beltrame 2024 [73]	J Endod. 2024. doi: 10.1016/j.joen.2024.02.016. PMID: 38428806.	No digital guidance component in the surgical workflow (No digital guidance component (CBCT measurement only))	October 2026
15	Ong 2020 [74]	Aust Dent J. 2020. doi: 10.1111/adj.12757. PMID: 32278321.	Case report or series below the five-case threshold (Fewer than five digitally guided transplants (1 of 6))	October 2026
16	Abella 2018	J Endod. 2018. Retracted; not cited in the reference list. PMID: 30154002.	Retracted publication	October 2026
17	Yu 2017 [75]	Int J Oral Maxillofac Surg. 2017. doi: 10.1016/j.ijom.2016.12.007. PMID: 28062250.	No digital guidance component in the surgical workflow (No digital guidance component)	October 2026
18	Yu 2013 [76]	Int J Oral Maxillofac Surg. 2013. doi: 10.1016/j.ijom.2013.08.019. PMID: 24148825.	No digital guidance component in the surgical workflow (No digital guidance component)	October 2026
