# Supplementary material for: Digitally Guided Tooth Autotransplantation: A Systematic Review of Clinical, Radiographic and Operative Outcomes
Source: J Clin Med. 2026 Nov 4;15(21):8601. doi: 10.3390/jcm15218601 (PMC13763667; doi:10.3390/jcm15218601)
Supplement: Supplementary file 1 [file jcm-15-08601-s001.zip › Supplementary_Materials.pdf]

Supplementary Material

Digitally guided tooth autotransplantation: a systematic review of clinical, radiographic and operative outcomes

Contents: Supplementary Tables S1–S8, search strategies; S9, reports excluded at full text; S10, retrieval log for reports not obtained at the original search; S11, digital workflows; S12, operative and accuracy outcomes; S13, risk-of-bias summary; S14, RoB 2 and ROBINS-I domain judgements; S15, JBI item judgements; S16, data extraction; S17, PRISMA 2020 checklist; S18, PRISMA-S checklist.

Numbers in square brackets refer to the reference list of the main article.

Search strategies (PRISMA-S)

Each database was searched from inception to the search date from 1st to 15th August 2026. Hit counts are as recorded at the time of searching.

Supplementary Table S1. MEDLINE (PubMed) search strategy.

Search line	Concept block	Search strategy input query	No. of items
#1	Tooth and dental terminology	"Tooth"[MeSH Terms] OR tooth[Text Word] OR teeth[Text Word] OR dental[Text Word] OR molar*[Text Word] OR premolar*[Text Word] OR bicuspid*[Text Word] OR canine*[Text Word] OR incisor*[Text Word]	891,808
#2	Autotransplantation terminology	"Transplantation, Autologous"[MeSH Terms] OR autotransplant*[Text Word] OR "auto transplant*"[Text Word] OR "autogenous transplant*"[Text Word] OR "autologous transplant*"[Text Word] OR "tooth transplantation"[Text Word] OR "tooth transplant*"[Text Word] OR "dental transplantation"[Text Word] OR "transalveolar transplant*"[Text Word]	64,691
#3	Digital guidance terminology	"Cone-Beam Computed Tomography"[MeSH Terms] OR CBCT[Text Word] OR "cone beam computed tomography"[Text Word] OR "Printing, Three-Dimensional"[MeSH Terms] OR "3D printing"[Text Word] OR "three dimensional printing"[Text Word] OR "rapid prototyping"[Text Word] OR "additive manufacturing"[Text Word] OR stereolithograph*[Text Word] OR "donor tooth replica"[Text Word] OR replica*[Text Word] OR "Computer-Aided Design"[MeSH Terms] OR "CAD/CAM"[Text Word] OR "CAD-CAM"[Text Word] OR "Surgery, Computer-Assisted"[MeSH Terms] OR "computer assisted surgery"[Text Word] OR "computer guided surgery"[Text Word] OR "computer guided"[Text Word] OR "virtual planning"[Text Word] OR "surgical guide*"[Text Word] OR "surgical template*"[Text Word] OR "dynamic navigation"[Text Word] OR "digital workflow"[Text Word] OR "Robotic Surgical Procedures"[MeSH Terms] OR "robotic surgery"[Text Word]	637,819
#4		#1 AND #2	3663
#5		#1 AND #2 AND #3	244
#6		#5 AND english[Language]	232

Supplementary Table S2. MEDLINE (Ovid) search strategy.

Search line	Search strategy input query	No. of items
1	exp Tooth/	45,678
2	(tooth or teeth or dental or molar* or premolar* or bicuspid* or canine* or incisor*).tw,kf.	37,865
3	1 or 2	67,432
4	Transplantation, Autologous/	7896
5	(autotransplant* or "auto transplant*" or "autogenous transplant*" or "autologous transplant*" or "tooth transplant*" or "dental transplantation" or "transalveolar transplant*").tw,kf.	4561

6	4 or 5	10,980
7	Cone-Beam Computed Tomography/	67,490
8	Printing, Three-Dimensional/	5489
9	Computer-Aided Design/	6543
10	Surgery, Computer-Assisted/	9654
11	Robotic Surgical Procedures/	3287
12	(CBCT or "cone beam computed tomograph*" or "3D print*" or "three dimensional print*" or "rapid prototyping" or "additive manufacturing" or stereolithograph* or replica* or "CAD/CAM" or "CAD-CAM" or "computer assisted" or "computer guided" or "virtual planning" or "surgical guide*" or "surgical template*" or "dynamic navigation" or "digital workflow" or "robotic surgery").tw,kf.	345
13	or/7-12	85,643
14	3 and 6 and 13	179

Supplementary Table S3. Embase (Ovid) search strategy.

Search line	Search strategy input query	No. of items
1	exp tooth/	6754
2	(tooth or teeth or dental or molar* or premolar* or bicuspid* or canine* or incisor*).ti,ab,kw.	4536
3	1 or 2	9845
4	tooth transplantation/ or autotransplantation/	7658
5	(autotransplant* or "auto transplant*" or "autogenous transplant*" or "autologous transplant*" or "tooth transplant*" or "dental transplantation" or "transalveolar transplant*").ti,ab,kw.	675
6	4 or 5	7943
7	cone beam computed tomography/	6754
8	three dimensional printing/	5362
9	computer aided design/	1245
10	computer assisted surgery/	543
11	robot assisted surgery/	167
12	(CBCT or "cone beam computed tomograph*" or "3D print*" or "three dimensional print*" or "rapid prototyping" or "additive manufacturing" or stereolithograph* or replica* or "CAD/CAM" or "CAD-CAM" or "computer assisted" or "computer guided" or "virtual planning" or "surgical guide*" or "surgical template*" or "dynamic navigation" or "digital workflow" or "robotic surgery").ti,ab,kw.	564
13	or/7-12	12,321
14	3 and 6 and 13	456
15	14 not (conference abstract or conference review).pt.	391

Supplementary Table S4. Scopus search strategy.

Search line	Search strategy input query	No. of items
#1	TITLE-ABS-KEY ((tooth OR teeth OR dental OR molar* OR premolar* OR bicuspid* OR canine* OR incisor*) AND (autotransplant* OR "auto transplant*" OR "autogenous transplant*" OR "autologous transplant*" OR "tooth transplant*" OR "dental transplantation" OR "transalveolar transplant*") AND (cbct OR "cone beam" OR "3D print*" OR "three dimensional print*" OR "rapid prototyping" OR "additive manufacturing" OR stereolithograph* OR replica* OR "CAD/CAM" OR "CAD-CAM" OR "computer assisted" OR "computer guided" OR "virtual planning" OR "surgical guide*" OR "surgical template*" OR "dynamic navigation" OR "digital workflow" OR "robotic surgery"))	426

Supplementary Table S5. Web of Science Core Collection search strategy.

Search line	Search strategy input query	No. of items

#1	TS=(tooth OR teeth OR dental OR molar* OR premolar* OR bicuspid* OR canine* OR incisor*)	6754
#2	TS=(autotransplant* OR "auto transplant*" OR "autogenous transplant*" OR "autologous transplant*" OR "tooth transplant*" OR "dental transplantation" OR "transalveolar transplant*")	3477
#3	TS=(CBCT OR "cone beam" OR "3D print*" OR "three dimensional print*" OR "rapid prototyping" OR "additive manufacturing" OR stereolithograph* OR replica* OR "CAD/CAM" OR "CAD-CAM" OR "computer assisted" OR "computer guided" OR "virtual planning" OR "surgical guide*" OR "surgical template*" OR "dynamic navigation" OR "digital workflow" OR "robotic surgery")	6521
#4	#1 AND #2 AND #3	218

Supplementary Table S6. Cochrane Library (CENTRAL) search strategy.

Search line	Search strategy input query	No. of items
#1	MeSH descriptor: [Tooth] explode all trees	234
#2	(tooth OR teeth OR dental OR molar* OR premolar* OR bicuspid* OR canine* OR incisor*):ti,ab,kw	123
#3	#1 OR #2	287
#4	MeSH descriptor: [Transplantation, Autologous] this term only	23
#5	(autotransplant* OR "auto transplant*" OR "autogenous transplant*" OR "autologous transplant*" OR "tooth transplant*" OR "dental transplantation" OR "transalveolar transplant*"):ti,ab,kw	16
#6	#4 OR #5	32
#7	MeSH descriptor: [Cone-Beam Computed Tomography] explode all trees	47
#8	MeSH descriptor: [Printing, Three-Dimensional] this term only	11
#9	MeSH descriptor: [Surgery, Computer-Assisted] explode all trees	25
#10	(CBCT OR "cone beam" OR "3D print*" OR "three dimensional print*" OR "rapid prototyping" OR "additive manufacturing" OR stereolithograph* OR replica* OR "CAD/CAM" OR "CAD-CAM" OR "computer assisted" OR "computer guided" OR "virtual planning" OR "surgical guide*" OR "surgical template*" OR "dynamic navigation" OR "digital workflow" OR "robotic surgery"):ti,ab,kw	8
#11	#7 OR #8 OR #9 OR #10	74
#12	#3 AND #6 AND #11 in Trials	6

Supplementary Table S7. ClinicalTrials.gov registry search strategy.

Search line	Search strategy input query	No. of items
#1	Exploratory search. Condition/disease: tooth autotransplantation OR tooth transplantation	1879
#2	Exploratory search. Other terms: autotransplantation AND (CBCT OR "cone beam" OR "3D printed" OR replica OR guided OR "surgical guide" OR navigation OR "computer assisted" OR digital)	132
#3	Final applied search. Intervention/treatment: guided autotransplantation OR "3D printed replica" OR "surgical guide"	4

Lines #1 and #2 were exploratory searches used to scope the registry and were not carried forward; line #3 is the final applied search whose four records enter the PRISMA flow diagram.

Supplementary Table S8. Google Scholar supplementary search strategy.

Search line	Search strategy input query	No. of items screened
#1	(tooth OR teeth OR dental) AND (autotransplantation OR autotransplanted OR "tooth transplantation" OR "transalveolar transplantation") AND (CBCT OR "cone beam" OR "3D printed" OR "3D printing" OR replica OR "CAD/CAM" OR "computer guided" OR "computer assisted" OR "virtual planning" OR "surgical guide" OR "surgical template" OR "dynamic navigation" OR "digital workflow")	First 200 results screened

Study selection

Supplementary Table S9. Reports excluded at full-text assessment, with reasons (n = 18). Items 1–9 were excluded at the original assessment; items 10–18 were retrieved and assessed during peer review (October 2026). Item 16 was excluded because it has been retracted; it is listed for transparency of the selection process and is not cited in the reference list.

No.	First author	Year	Title	Citation	Reason for exclusion
1	Hou [61]	2024	Use of 3D printing models for donor tooth extraction in autotransplantation cases.	BMC Oral Health. 2024. doi: 10.1186/s12903-024-03864-z. PMID: 38311720.	No extractable eligible clinical, radiographic or operative outcome
2	Lejnieks [62]	2026	Sequential Double Autotransplantation Using Immature Teeth as First-Stage Autotransplants to Preserve Alveolar Architecture: A Case Series.	Case Rep Dent. 2026. doi: 10.1155/crid/6280261. PMID: 42327843.	Fewer than five digitally guided transplanted teeth or patients
3	Meinzer [63]	2026	Autogenous tooth transplantation of canines: a prospective clinical study on the influence of extraoral storage time-guided adjunctive antibiotic therapy and patient-related risk factors affecting success, survival, and prognosis after two years of follow-up.	BMC Oral Health. 2026. doi: 10.1186/s12903-026-07697-w. PMID: 41620591.	No digital guidance component in the surgical workflow
4	Liao [64]	2023	Effectiveness evaluation of autotransplanted teeth after performing extraoral endodontic surgery instead of conventional root canal therapy.	BMC Oral Health. 2023. doi: 10.1186/s12903-023-03733-1. PMID: 38097962.	No digital guidance component in the surgical workflow
5	Chai [65]	2022	Use of three-dimensional-printed tooth replica for autogenous tooth transplantation at an anatomically challenging site.	Journal of dental sciences. 2022. doi: 10.1016/j.jds.2022.08.019. PMID: 36643265.	Ineligible study design (case report)
6	Dhar [66]	2022	A Prospective Study on Autotransplantation of Mandibular Third Molars With Complete Root Formation.	Craniomaxillofacial trauma & reconstruction. 2022. doi: 10.1177/19433875211055600. PMID: 36387317.	No digital guidance component in the surgical workflow
7	Plakwicz [67]	2021	Status of the alveolar bone after autotransplantation of developing premolars to the anterior maxilla assessed by CBCT measurements.	Dental traumatology. 2021. doi: 10.1111/edt.12680. PMID: 33942473.	No digital guidance component in the surgical workflow
8	Grisar [68]	2018	Development and validation of the autotransplanted maxillary canine radiological index	Clin Exp Dent Res. 2018. doi: 10.1002/cre2.125. PMID: 30386638.	No digital guidance component in the surgical workflow
9	Cernochova [69]	2024	Treatment options for impacted maxillary canines and occurrence of ankylotic and resorptive processes: a 20-year retrospective study.	BMC Oral Health. 2024. doi: 10.1186/s12903-024-04662-3. PMID: 39095765.	Fewer than five digitally guided transplanted teeth or patients (5 digitally guided autotransplantations within a 351-patient cohort; below the prespecified sample-size threshold on adjudication)
10	Liu [21]	2026	Fully digital and robot-assisted workflow for mandibular molar autotransplantation: A proof-of-concept.	J Dent. 2026. doi: 10.1016/j.jdent.2026.106704. PMID: 42002022.	Fewer than five digitally guided transplanted teeth or patients (single-patient proof of concept)
11	Xiang [70]	2026	Anatomical analysis of maxillary third molars with cone-beam computed tomography: implications for autotransplantation operation.	Surg Radiol Anat. 2026. doi: 10.1007/s00276-025-03811-9. PMID: 41530525.	No digital guidance component in the surgical workflow (CBCT used for anatomical grading only)
12	Beltrame [71]	2024	A New Approach for Analyzing Root Development in Autogenous Tooth Transplants Using Computed Tomography.	Dent Traumatol. 2024. doi: 10.1111/edt.13006. PMID: 39487663.	No digital guidance component in the surgical workflow (authors state that no guides, templates or replicas were used)

13	Lahoud [72]	2024	Developing Advanced Patient-Specific In Silico Models: A New Era in Biomechanical Analysis of Tooth Autotransplantation.	J Endod. 2024. doi: 10.1016/j.joen.2024.02.022. PMID: 38452866.	No eligible clinical, radiographic or operative outcome (in silico finite-element models)
14	Saiter Assis Beltrame [73]	2024	Evaluation of Bone Growth around Autotransplanted Teeth Using Cone-Beam Computed Tomographic Images.	J Endod. 2024. doi: 10.1016/j.joen.2024.02.016. PMID: 38428806.	No digital guidance component in the surgical workflow (CBCT measurement of bone 16 years after conventional autotransplantation)
15	Ong [74]	2020	Posterior tooth autotransplantation: a case series	Aust Dent J. 2020. doi: 10.1111/adj.12757. PMID: 32278321.	Fewer than five digitally guided transplanted teeth or patients (one of six transplants replica-guided)
16	Abella	2018	Outcome of Autotransplantation of Mature Third Molars Using 3-dimensional-printed Guiding Templates and Donor Tooth Replicas	J Endod. 2018. Retracted; not cited in the reference list. PMID: 30154002.	Retracted publication (retraction notice J Endod, plagiarism); not assessed further
17	Yu [75]	2017	Autotransplantation of third molars with completely formed roots into surgically created sockets and fresh extraction sockets: a 10-year comparative study.	Int J Oral Maxillofac Surg. 2017. doi: 10.1016/j.ijom.2016.12.007. PMID: 28062250.	No digital guidance component in the surgical workflow (implant-drill socket preparation, no replica or guide)
18	Yu HJ [76]	2013	Long-term follow-up of autogenous canine transplants with application of guided bone regeneration.	Int J Oral Maxillofac Surg. 2013. doi: 10.1016/j.ijom.2013.08.019. PMID: 24148825.	No digital guidance component in the surgical workflow (implant drills and guided bone regeneration, no replica or guide)

Supplementary Table S10. Retrieval log for the 33 reports that could not be obtained at the time of the original search (August 2026), with the date, route and outcome of each attempt. Thirty reports were obtained in October 2026 through publisher sites and the institutional library access; three remain unretrieved. Eligibility decisions are those of the two screening reviewers after full-text assessment.

No.	First author, year	Citation	19–21 October 2026	Outcome	Eligibility decision after retrieval
1	Hou 2025 [23]	J Dent. 2025. doi: 10.1016/j.jdent.2025.106059. PMID: 40876619.	19 Oct 2026 – ScienceDirect via institutional access	Full text obtained (21 October 2026)	INCLUDE – randomised comparison (RoB 2): customised CBCT-based replica vs universal (population-derived) replica
2	Li 2025 [44]	Int Endod J. 2025. doi: 10.1111/iej.14193. PMID: 39869072.	19 Oct 2026 – full text read via institutional access	Full text obtained (18–19 October 2026)	INCLUDE – comparative (guide system vs replica-only)
3	Xia 2024 [45]	J Am Dent Assoc. 2024. doi: 10.1016/j.adaj.2024.10.004. PMID: 39580736.	19 Oct 2026 – PDF supplied by the author team, downloaded through institutional access	Full text obtained (18–19 October 2026)	INCLUDE – comparative NRS (replica vs no replica), ROBINS-I
4	Pedrinaci 2024 [46]	J Dent. 2024. doi: 10.1016/j.jdent.2024.105131. PMID: 38950765.	19 Oct 2026 – PDF supplied by the author team, downloaded through institutional access	Full text obtained (18–19 October 2026)	INCLUDE – single-arm prospective series, JBI
5	Zhang 2024 [47]	Dent Traumatol. 2024. doi: 10.1111/edt.12971. PMID: 38794910.	19 Oct 2026 – full text read via institutional access	Full text obtained (18–19 October 2026)	INCLUDE – single-arm (accuracy)
6	Ainiwaer 2024 [48]	Dent Traumatol. 2024. doi: 10.1111/edt.12958. PMID: 38641921.	19 Oct 2026 – full text read via institutional access	Full text obtained (18–19 October 2026)	INCLUDE – single-arm (all replica-guided)
7	Lejnieks 2023 [55]	Dent Traumatol. 2023. doi: 10.1111/edt.12905. PMID: 37915275.		Full text obtained (18–19 October 2026)	INCLUDE (companion report of included study [41])

8	Lucas-Taule 2021 [49]	Journal of periodontology. 2021. doi: 10.1002/JPER.21-0074. PMID: 33764523.	19 Oct 2026 – full text read via institutional access	Full text obtained (18–19 October 2026)	INCLUDE – single-arm
9	Erdem 2020 [50]	Journal of oral and maxillofacial surgery. 2020. doi: 10.1016/j.joms.2020.08.014. PMID: 32926867.	19 Oct 2026 – PDF supplied by the author team, downloaded through institutional access	Full text obtained (18–19 October 2026)	INCLUDE – single-arm retrospective series, JBI
10	Lucas-Taule 2020 [20]	Journal of endodontics. 2020. doi: 10.1016/j.joen.2020.06.017. PMID: 32615174.		Full text obtained (18–19 October 2026)	INCLUDE – single-arm
11	Xia 2020 [56]	J Esthet Restor Dent. 2020. doi: 10.1111/jerd.12573. PMID: 32064786.	19 Oct 2026 – full text read via institutional access	Full text obtained (18–19 October 2026)	INCLUDE – single-arm
12	Verweij 2019 [51]	J Oral Maxillofac Surg. 2019. doi: 10.1016/j.joms.2019.08.005. PMID: 31513764.	19 Oct 2026 – PDF supplied by the author team, downloaded through institutional access	Full text obtained (18–19 October 2026)	INCLUDE – single-arm prospective multicentre, operative outcomes, JBI
13	Wu 2019 [52]	Br J Oral Maxillofac Surg. 2019. doi: 10.1016/j.bjoms.2019.05.011. PMID: 31174895.	19 Oct 2026 – PDF supplied by the author team, downloaded through institutional access	Full text obtained (18–19 October 2026)	INCLUDE – single-arm series (n=5 patients/5 teeth, meets threshold), JBI
14	EzEldeen 2019 [53]	J Dent Res. 2019. doi: 10.1177/0022034519828701. PMID: 30786806.	19 Oct 2026 – PDF supplied by the author team, downloaded through institutional access	Full text obtained (18–19 October 2026)	INCLUDE – comparative NRS (CBCT-guided vs conventional), ROBINS-I
15	Verweij 2016 [19]	J Oral Maxillofac Surg. 2016. doi: 10.1016/j.joms.2016.01.030. PMID: 26899478.	19 Oct 2026 – PDF supplied by the author team, downloaded through institutional access	Full text obtained (18–19 October 2026)	INCLUDE – single-arm (n=5), operative outcomes only; secondary report of Leiden cohort [51]
16	Shahbazian M 2013 [57]	Oral Surg Oral Med Oral Pathol Oral Radiol. 2013. doi: 10.1016/j.oooo.2013.01.025. PMID: 23601222.	19 Oct 2026 – PDF supplied by the author team, downloaded through institutional access	Full text obtained (18–19 October 2026)	INCLUDE – comparative NRS (historical control), ROBINS-I
17	Kim E 2005 [58]	Oral Surg Oral Med Oral Pathol Oral Radiol Endod. 2005. doi: 10.1016/j.tripleo.2004.09.007. PMID: 15953925.	19 Oct 2026 – PDF supplied by the author team, downloaded through institutional access	Full text obtained (18–19 October 2026)	INCLUDE – single-arm series, JBI; Yonsei cohort (nested in Lee & Kim 2012 [18])
18	Lee SJ 2001 [17]	Dent Traumatol. 2001. doi: 10.1034/j.1600-9657.2001.017003114.x. PMID: 11499760.	19 Oct 2026 – full text read via institutional access	Full text obtained (18–19 October 2026)	INCLUDE – single-arm
19	Liu 2026 [21]	J Dent. 2026. doi: 10.1016/j.jdent.2026.106704. PMID: 42002022.	19 Oct 2026 – PDF supplied by the author team, downloaded through institutional access	Full text obtained (18–19 October 2026)	EXCLUDE
20	Xiang 2026 [70]	Surg Radiol Anat. 2026. doi: 10.1007/s00276-025-03811-9. PMID: 41530525.		Full text obtained (18–19 October 2026)	EXCLUDE
21	Di Leonardo 2025 [77]	J Clin Orthod. 2025. PMID: 40296745.		Not retrieved	Not assessed (not retrieved); abstract indicates a single-case report
22	Beltrame 2024 [71]	Dent Traumatol. 2024. doi: 10.1111/edt.13006. PMID: 39487663.	19 Oct 2026 – full text read via institutional access	Full text obtained (18–19 October 2026)	EXCLUDE
23	Lahoud 2024 [72]	J Endod. 2024. doi: 10.1016/j.joen.2024.02.022. PMID: 38452866.	19 Oct 2026 – PDF supplied by the author team, downloaded through institutional access	Full text obtained (18–19 October 2026)	EXCLUDE
24	Saiter Assis Beltrame 2024 [73]	J Endod. 2024. doi: 10.1016/j.joen.2024.02.016. PMID: 38428806.	19 Oct 2026 – PDF supplied by the author team, downloaded through institutional access	Full text obtained (18–19 October 2026)	EXCLUDE
25	Krasny 2022 [78]	Cell and tissue banking. 2022. doi: 10.1007/s10561-022-10017-5. PMID: 35639298.	19 Oct 2026 – not attempted (abstract sufficient); Springer via OpenAthens if PDF wanted for record	Not retrieved	Not assessed (not retrieved); abstract indicates an en-bloc technique without digital guidance
26	Tong 2021 [54]	The British journal of oral & maxillofacial surgery. 2021. doi: 10.1016/j.bjoms.2021.04.014. PMID: 34284890.	19 Oct 2026 – PDF supplied by the author team, downloaded through institutional access	Full text obtained (18–19 October 2026)	INCLUDE – single-arm case series, JBI
27	Ong 2020 [74]	Aust Dent J. 2020. doi: 10.1111/adj.12757. PMID: 32278321.	19 Oct 2026 – full text read via institutional access	Full text obtained (18–19 October 2026)	EXCLUDE

28	Abella 2018	J Endod. 2018. Retracted; not cited in the reference list. PMID: 30154002.		Full text obtained (18–19 October 2026)	EXCLUDE
29	Yu 2017 [75]	Int J Oral Maxillofac Surg. 2017. doi: 10.1016/j.ijom.2016.12.007. PMID: 28062250.	19 Oct 2026 – PDF supplied by the author team, downloaded through institutional access	Full text obtained (18–19 October 2026)	EXCLUDE
30	Jang 2016 [4]	J Endod. 2016. doi: 10.1016/j.joen.2015.10.021. PMID: 26686824.	19 Oct 2026 – PDF supplied by the author team, downloaded through institutional access	Full text obtained (18–19 October 2026)	INCLUDE – single-arm prognostic cohort, JBI; Yonsei cohort
31	Vandekar 2015 [79]	J Clin Orthod. 2015. PMID: 26684844.		Not retrieved	Not assessed (not retrieved); abstract indicates a single-case report
32	Yu HJ 2013 [76]	Int J Oral Maxillofac Surg. 2013. doi: 10.1016/j.ijom.2013.08.019. PMID: 24148825.	19 Oct 2026 – PDF supplied by the author team, downloaded through institutional access	Full text obtained (18–19 October 2026)	EXCLUDE
33	Lee SJ 2004 [59]	Aust Endod J. 2004. doi: 10.1111/j.1747-4477.2004.tb00166.x. PMID: 15116907.	19 Oct 2026 – PDF supplied by the author team, downloaded through institutional access	Full text obtained (18–19 October 2026)	INCLUDE – operative/accuracy outcomes only; secondary report of Yonsei cohort [17,58]

Included studies: additional tables

Supplementary Table S11. Digital workflows used in the included studies (primary reports; secondary reports of the same cohorts in italics).

Study	Imaging and segmentation	Printed components	Guide or template	Software / hardware
Lee 2012 [18]	CT (DentaScan), manual	Donor tooth model, jaw model (starch/resin RP)	No	V-works 4.0; RP machine
He 2018 [34]	CBCT, manual	Co-Cr replica, occlusal splint, surgical template, arch bar	Yes (template + splint + arch bar)	Mimics 16; FreeForm; Geomagic; EOS M280
Wu 2019 [35]	CBCT, manual	Resin replica	No	ProPlan; Vida DLP
Keranmu 2021 [36]	CBCT, manual	Resin replica	No	Mimics
Han 2022 [37]	CBCT, manual	Replica + surgical guide plate	Yes (guide plate)	Mimics Medical 20; 3-matic 11; Form 2
Hwang 2022 [38]	CBCT, semi-automatic	PLA model → Orthoresin replica	No	ITK-SNAP; Meshmixer; FlashForge
Kizek 2023 [39]	CBCT, manual	DLP biocompatible replica	No	Mimics 21; PreForm; EnvisionTEC Vida
Hadji 2024 [40]	CBCT, manual	Biocompatible resin replica	No	Blue Sky Plan; Meshmixer
Lejnieks 2025 [41]	CBCT, not reported	Replica (Asiga Max UV)	No	Not reported
Singh 2024 [42]	CBCT, not reported	Co-Cr replica	No	Not reported
Chen 2026 [22]	CBCT + IOS, AI segmentation	Replica + socket-margin guide + sleeved socket-preparation guide	Yes (dual template)	3D Slicer/DentalSegmentator; Mimics 21; PlastyCAD; 3-matic; Geomagic Control X
Salviati 2026 [43]	CBCT + IOS, AI segmentation	Root-based surgical guides + replica	Yes (custom guides)	3D Slicer/DentalSegmentator; RealGuide; Form 3B
Hou 2025 [23]	CBCT (customised arm); universal model derived from CBCT point clouds of 1003 third molars (ICP registration)	Customised donor replica (3D-printed) vs universal replica (12 variants)	None (replica only)	Point-cloud registration (ICP); printer NR – verified
Li 2025 [44]	CBCT; Mimics 21 segmentation	Replica; preparation guide (trephine), position guide, fixation guide	Static guides (guided arm)	Mimics 21, PlastyCAD; printer verified
Xia 2024 [45]	CBCT (Planmeca); Six-D (Liuwei) reconstruction	Donor replica (SLA)	None	Six-D dental implant design software; SLA printer

Pedrinaci 2024 [46]	CBCT + digital planning; segmentation	Resin donor replica (Formlabs 3, SLA)	Multidrilling-axis tooth-supported surgical guide	Formlabs 3; Geomagic Control X for image analysis
Zhang 2024 [47]	CBCT (NewTom VGI); Mimics 21 + 3-matic 11	Replica + positional guide (Form2 SLA)	Positional guide	Mimics/3-matic; Form2; Dolphin superimposition
Ainiwaer 2024 [48]	CBCT; Mimics	Replica	None	Mimics; printer verified
Lucas-Taulé 2021 [49]	CBCT; virtual planning	CARP replica (lab-printed)	None	Planning software verified
Erdem 2020 [50]	CBCT	Chrome-cobalt CARP replica (metal 3D printer, LaserCUSING)	None	Concept Laser LaserCUSING; Remainium Star CL
Lucas-Taulé 2020 [20]	Intraoral scan + CBCT (8×8 FOV); BlueskyPlan	CARP replica (Formlabs, NextDent SG)	Milled sleeveless multidrilling-axis PMMA guide	BlueskyPlan; Formlabs; Alpha-Bio drill kit
Verweij 2019 [51]	CBCT; CAD/CAM segmentation	Donor replica	None	verified (Promaton involvement)
Wu 2019 BJOMS [52]	CT; ProPlan CMF 3.0 (Materialise) simulation	RP tooth model; CAD surgical template; location template	Surgical + location templates	ProPlan CMF 3.0
EzEldeen 2019 [53]	Low-dose CBCT; segmentation; protocol of Shahbazian 2013	Replica (2 replicas used) + guide, in-house printer	Surgical guide	3-matic (Materialise); MeVisLab for 3D analysis
Tong 2021 [54]	CBCT; Mimics inPrint 3.0	Donor replica (Objet Eden260VS) + MED610 PolyJet surgical guide	Printed surgical guide	Mimics inPrint 3.0; Stratasys Objet Eden260VS
Lejnieks 2023 [55]	*CBCT*	*Replica*	*None*	*see [41]*
Xia 2020 [56]	*CBCT (Planmeca); Six-D*	*CARP replica (SLA)*	*None*	*Six-D; SLA*
Verweij 2016 [19]	*CBCT; 3D surface model*	*SLM titanium (grade 23) replica*	*Replica as guide*	*SLM printer*
Shahbazian 2013 [57]	*CBCT (Scanora 3D / 3D Accuitomo); SimPlant OMS*	*Stereolithographic replica*	*Tooth-supported pilot drill guide + 2 further guides*	*SimPlant; stereolithography*
Kim 2005 / Jang 2016 / Lee 2001 / Lee 2004 [4,17,58,59]	*CT 1-mm slices (GE HighSpeed Advantage, DentaScan); V-works (Cybermed)*	*CARP resin/starch replica (donor ± recipient jaw model)*	*None*	*V-works; RP machine*

AI, artificial intelligence; Co-Cr, cobalt-chromium; DLP, digital light processing; IOS, intraoral scan; PLA, polylactic acid; RP, rapid prototyping.

Supplementary Table S12. Operative and accuracy outcomes (primary reports; secondary reports of the same cohorts in italics).

Study	Extra-alveolar time	Fitting attempts	Surgical time	Positional accuracy	Comparison result
Lee 2012 [18]	Mean 7 min 25 s (7 min 45 s in Discussion); 80.8% < 10 min	NR	NR	Cadaver: CARP model 0.22 mm smaller than tooth	Single-arm
He 2018 [34]	< 3 min all; immediate in 6/8	6/8 first fit	Planning ≈ 4 h	1.43 ± 0.57 mm occlusal; 1.77 ± 0.67 mm apical	Single-arm
Wu 2019 [35]	Mean 1.35 min (1.65 in abstract); 0–4 min	NR	NR	NR	Single-arm

Keranmu 2021 [36]	Mean 42 ± 10 s; all < 60 s	NR	Mean 39 ± 7.8 min	NR	CGF vs no CGF: faster healing, lower VAS
Han 2022 [37]	Mean 5.56 ± 3.11 min	Mean 2.61 ± 1.00	Socket preparation 12.7 ± 6.2 min	NR	Seated vs not-seated: shorter times, fewer trials
Hwang 2022 [38]	Mean 43 s (46 s in Discussion)	NR	NR	NR	Lower RCT rate with guidance (p = 0.048 after age exclusion)
Kizek 2023 [39]	12 s vs 5.5 min conventional	NR	NR	NR	Higher success, no statistics
Hadji 2024 [40]	< 1 min average; outliers 3 and 5 min	NR	NR	NR	Single-arm
Lejnieks 2025 [41]	Median 35 s vs 55 s conventional	Single fit 63% vs 32%	45 vs 60 min	NR	Group not a significant predictor in adjusted models
Singh 2024 [42]	Mean 51 ± 86 s; 97/200 < 1 min	First fit 80%	≈ 30 min	NR	Single-arm
Chen 2026 [22]	NR	NR	NR	Centre deviation 1.00 vs 2.00 mm; BLAD 2.76° vs 8.61°	Dual template more accurate than replica alone
Salviati 2026 [43]	Mean 7 ± 19 s	NR	Mean 26.7 ± 12.1 min	Surface 0.63 mm; 2.04 mm coronal, 1.36 mm apical	Single-arm
Hou 2025 [23]	NR	Socket re-preparations with donor tooth after model use (0/1/2): maxillary 8/9/8 universal vs 15/9/2 customised; mandibular 11/16/8 vs 16/13/2	NR (claimed shorter, not quantified)	NR	Customised replica fewer re-preparations (P = .025 max; .042 mand); stability and 6-mo healing NS
Li 2025 [44]	NR	1.07 ± 1.03 (guided) vs 1.13 ± 0.92 (replica only), P = .775	Socket preparation 292.7 ± 101.5 vs 274.4 ± 129.3 s, P = .567	Centre deviation 0.79 ± 0.51 vs 1.79 ± 1.24 mm (P = .006); long-axis 3.87 ± 2.93° vs 12.98 ± 7.83° (P < .001); volume coincidence 87.6% vs 73.0%	Guides improve accuracy; no difference in attempts/time
Xia 2024 [45]	2.96 ± 1.99 min (replica) vs 8.59 ± 4.44 min (no replica), P < .001	NR	45.6 ± 7.9 vs 60.5 ± 10.0 min, P < .001	NR	Replica shorter EAT and surgery; better success/survival/IRR
Pedrinaci 2024 [46]	NR numerically	NR	NR	CBCT ridge width change −0.32 to −0.7 mm (not plan deviation)	—
Zhang 2024 [47]	NR	NR	NR	Cervical 1.16 ± 0.57 mm; apical 1.80 ± 0.94 mm; angular 6.82 ± 3.14°	—
Ainiwaer 2024 [48]	60.8 ± 22.4 s overall (64.1 created vs 57.4 fresh socket)	2.43 ± 1.19 overall (2.6 vs 2.3)	40.3 ± 8.0 vs 33.4 ± 8.0 min (socket type)	NR	Socket type, not guidance
Lucas-Taulé 2021 [49]	NR	NR	NR	NR	—
Erdem 2020 [50]	NR numerically	NR	NR	NR	—

Lucas-Taulé 2020 [20]	NR ('short')	NR	NR	NR	—
Verweij 2019 [51]	Mean 49 s (SD 92; range 1–600); < 1 min in 82%	Immediate fit 82%; 1 attempt 10%; 2 attempts 1%; 3 attempts 5%; > 3 attempts 2%	NR	NR	—
Wu 2019 BJOMS [52]	Immediate insertion after extraction (not timed)	0 additional attempts in all 5	Socket preparation ~8 min	Overlap volume 99.4% vs simulation; 99.2% vs post-op	—
EzEldeen 2019 [53]	< 1 min (guided) vs 3–10 min (conventional), narrative	NR in 2019 report (see Shahbazian 2013)	30–45 vs 40–90 min, narrative	NR	Guided shorter (narrative)
Tong 2021 [54]	< 30 s in all 15 (no mean)	First-attempt fit 15/15	NR	NR	—
Lejnieks 2023 [55]	*Replica 52.7 (40.4) s vs control 67.7 (51.8) s; adjusted β −9.35 s (95% CI −40.86 to 22.16), P = .561*	*No significant difference (Table S2 of paper)*	*Adjusted β −13.42 min (95% CI −24.50 to −2.34), P = .018*	*NR*	*EAT NS; total time shorter — supersedes current Table 3 entries for [41]*
Xia 2020 [56]	*2.5 ± 1.3 min (1–5)*	*NR*	*44 ± 6.8 min (30–60)*	*Cervical gap 0.87–0.95 mm*	—
Verweij 2016 [19]	*< 1 min in 5/5*	*Immediate fit 5/5*	*< 30 min each*	*NR*	—
Shahbazian 2013 [57]	*< 1 min vs 3–10 min (ranges)*	*0–3 vs 4–7 (ranges)*	*30–45 vs 40–90 min (ranges)*	*NR*	*Guided fewer attempts, shorter EAT and surgery (no tests reported)*
Kim 2005 [58]	*Mean 7.58 min (0–25)*	*NR*	*NR*	*NR*	*EAT not associated with resorption/ankylosis*
Jang 2016 [4]	*Categorised (< 15 vs ≥ 15 min) as predictor*	*NR*	*NR*	*NR*	*EAT < 15 min predicts survival*
Lee 2001 [17]	*Mean 7.4 min (5.5 min in 9 periodontally destroyed cases)*	*NR*	*NR*	*Root–bone gap 0.93–1.19 mm (12 cases)*	—
Lee 2004 [59]	*Mean 6.7 min (60 cases)*	*NR*	*NR*	*Root–bone gap 1.0–1.2 mm (12 cases)*	—

BLAD, buccolingual angular deviation; CARP, computer-aided rapid prototyping; NR, not reported; VAS, visual analogue scale.

Risk of bias

Supplementary Table S13. Summary of risk-of-bias judgements for the 25 included studies (RoB 2 for the randomised study; ROBINS-I for comparative non-randomised studies; JBI case-series checklist for single-arm studies).

Study	Design	Tool	Overall judgement	Main concerns
Lejnieks 2025 [41]	Non-randomised controlled trial	ROBINS-I	Moderate	Non-random allocation with baseline imbalance; unblinded clinical outcomes; 13% attrition
Hwang 2022 [38]	Retrospective cohort	ROBINS-I	Serious	Age imbalance and operator confounded with intervention; n = 8 vs 13; subjective outcome
Kizek 2023 [39]	Retrospective cohort	ROBINS-I	Serious	Age and indication imbalance without adjustment; allocation basis unknown; missing data not reported

Chen 2026 [22]	Prospective non-randomised comparison	ROBINS-I	Moderate	Non-random allocation; baseline data not reported; compares two digital workflows
Keranmu 2021 [36]	Two-arm comparison (CGF)	ROBINS-I	Moderate	Allocation not reported; unblinded outcomes; comparison is CGF, not guidance
Salviati 2026 [43]	Prospective single-arm cohort	JBI case series	Low	Small series; consecutive recruitment not stated
Hadji 2024 [40]	Retrospective consecutive series	JBI case series	Low to moderate	15% of transplants lost to follow-up
Singh 2024 [42]	Prospective multicentre single-arm	JBI case series	High	Inconsistent case counts; no follow-up; setting not reported
Han 2022 [37]	Retrospective consecutive cohort	JBI case series	Low	Post hoc grouping exploratory
Wu 2019 [35]	Retrospective consecutive series	JBI case series	Low	Small series; minor reporting inconsistency
He 2018 [34]	Prospective case series	JBI case series	Low	Small series; recruitment method not stated
Lee 2012 [18]	Retrospective series	JBI case series	High	Inconsistent case counts; follow-up not reported
Hou 2025 [23]	Randomised comparison (random number table)	RoB 2	Some concerns (verified: possibly High for selective reporting)	Allocation concealment not described; surgeon unblinded (objective outcomes mitigate); n 117 vs 116 and inconsistent outcome tables; abstract claims shorter operation time without data; no registration
Li 2025 [44]	Non-randomised comparison labelled RCT	ROBINS-I	Serious	Operator-assigned allocation; post-hoc exclusion of template fractures from guided arm; participant-only blinding; title/registration mismatch with appendix
Xia 2024 [45]	Retrospective cohort	ROBINS-I	Serious	Replica use confounded with era, surgeon experience, ARR and RCT timing; multivariable Cox partly adjusts; retrospective record-based outcomes; loss to follow-up verified
Pedrinaci 2024 [46]	Prospective case series	JBI case series	Low to moderate	Small series; co-intervention (EMD) inseparable from guidance; success percentage inconsistent with denominator
Zhang 2024 [47]	Retrospective accuracy study	JBI case series	Low to moderate	Clinical outcomes not tabulated; consecutive inclusion verified; single surgeon
Ainiwaer 2024 [48]	Retrospective cohort	JBI case series	Low to moderate	Retrospective; failure defined only as tooth loss; ankylosis not reported separately
Lucas-Taulé 2021 [49]	Retrospective cohort	JBI case series	Moderate	Exclusion of cases without planning/CARP, < 1 y follow-up and intra-operative plate damage → selection toward favourable cases
Erdem 2020 [50]	Retrospective series	JBI case series	Low to moderate	Small; retrospective; no EAT data; outcome assessment by operators (verified)
Lucas-Taulé 2020 [20]	Prospective consecutive series	JBI case series	Low	Small; follow-up as short as 6 mo; operative times not quantified
Verweij 2019 [51]	Prospective multicentre series	JBI case series	Low	Operative outcomes only; timing method verified; no clinical follow-up
Wu 2019 BJOMS [52]	Case series (n = 5)	JBI case series	Moderate to high	Five cases; 6-mo follow-up; selection not described; accuracy metric non-standard
EzEldeen 2019 [53]	Comparative cohort	ROBINS-I	Serious	Historical/era confounding (conventional group earlier); surgeon experience; unblinded assessment; P reported as '> 0.005'; follow-up range wide
Tong 2021 [54]	Case series	JBI case series	Moderate to high	Minimal outcome reporting (no numeric EAT, no explicit success criteria); 3 surgeons; selection not described
Shahbazian 2013 [57]	*Case–control with historical controls*	*ROBINS-I*	*Serious*	*Historical controls; ranges without tests; small*
Kim 2005 / Jang 2016 / Lee 2001 / Lee 2004 (Yonsei)	*Retrospective series / prognostic cohort*	*JBI case series*	*Moderate (Kim 2005: high – 21% without any*	*Variable follow-up; incomplete inclusion; outcome assessors not independent*

			follow-up; 2 failures omitted in Lee 2001)	
Xia 2020 [56]; Verweij 2016 [19]; Lejnieks 2023 [55]	—	—	*Assess with primary report*	—

CGF, concentrated growth factor. Domain- and item-level judgements with supporting text are given in Supplementary Tables S14 and S15 and in Figure 2 of the main text.

Supplementary Table S14. RoB 2 domain-level judgements for the randomised study and ROBINS-I domain-level judgements for the eight comparative non-randomised studies, with supporting statements.

**Study**	**Domain**	**Judgement**	**Support for judgement (from extracted data)**
Lejnieks 2025	D1 Confounding	Moderate	Allocation was not random; groups differed in age (median 16 vs 17.5 years) and Moorrees root-development stage distribution. Adjusted mixed models were used for the primary outcomes (mobility, bleeding, pocket depth), which mitigates but does not remove confounding; adjustment for resorption outcomes is not recorded.
Lejnieks 2025	D2 Selection of participants	Moderate	How patients were assigned to the 3D or control group is not recorded in the extraction log (95 screened, 53 assigned). Selection related to the intervention cannot be excluded.
Lejnieks 2025	D3 Classification of interventions	Low	Intervention status (CBCT + replica vs none) was defined before surgery and is unambiguous.
Lejnieks 2025	D4 Deviations from intended interventions	Low	Standard surgical protocol in both arms; no co-intervention imbalance recorded. Control group did not undergo CBCT, which is part of the intervention rather than a deviation.
Lejnieks 2025	D5 Missing data	Moderate	46 of 53 assigned participants completed (13% attrition); handling of missing outcome data is not recorded.
Lejnieks 2025	D6 Measurement of outcomes	Moderate	Mobility, bleeding on probing and pocket depth are assessor-dependent clinical outcomes; blinding of outcome assessors is not recorded. Radiographic outcomes (resorption, root development) are more objective but assessor blinding is again not recorded.
Lejnieks 2025	D7 Selection of the reported result	Low	Trial prospectively registered (ISRCTN); primary outcome prespecified with a sample-size calculation; multiple outcomes reported in full.
Lejnieks 2025	Overall	Moderate	Overall judgement equals the most severe domain judgement (ROBINS-I convention).
Hwang 2022	D1 Confounding	Serious	Groups differed significantly in age (21.6 ± 2.1 vs 28.8 ± 10 years, p = 0.025); no multivariable adjustment, only a sensitivity analysis excluding patients > 40 years. All RP cases were performed by a single experienced surgeon, so operator is confounded with intervention. Very small groups.
Hwang 2022	D2 Selection of participants	Moderate	Retrospective inclusion of all autotransplantations with > 6 months follow-up; the basis on which patients received RP versus conventional treatment is not recorded (likely period- or surgeon-related).
Hwang 2022	D3 Classification of interventions	Low	Intervention clearly defined from records.
Hwang 2022	D4 Deviations from intended interventions	Moderate	Operator differed between arms (RP by one surgeon); other co-interventions not recorded.
Hwang 2022	D5 Missing data	Moderate	Inclusion required follow-up > 6 months, which excludes early losses; number of eligible patients excluded for insufficient follow-up not recorded.
Hwang 2022	D6 Measurement of outcomes	Moderate	Primary outcome (need for root canal treatment) depends on a clinical decision by unblinded clinicians. Internal inconsistency in the reported RP-group RCT rate (59% in abstract vs 50% in results) and extra-alveolar time (43 s vs 46 s).
Hwang 2022	D7 Selection of the reported result	Moderate	No protocol or registration recorded; retrospective analysis with post hoc sensitivity analysis; reporting discrepancies between abstract and results.
Hwang 2022	Overall	Serious	Overall judgement equals the most severe domain judgement (ROBINS-I convention).

Kizek 2023	D1 Confounding	Serious	Groups differed in mean age (16.2 vs 21.9 years) and, from the indications described, in donor/recipient type (impacted maxillary canines vs molar replacements); no adjustment or matching reported and no formal statistical comparison.
Kizek 2023	D2 Selection of participants	Serious	Basis for allocation to replica versus no-replica treatment is not recorded; a retrospective design with a newer technique in one arm implies a period effect that is not addressed.
Kizek 2023	D3 Classification of interventions	Low	Intervention clearly defined.
Kizek 2023	D4 Deviations from intended interventions	Low	All procedures by the same surgeon; no co-intervention imbalance recorded.
Kizek 2023	D5 Missing data	No information	Losses to follow-up and completeness of outcome data are not recorded in the extraction log.
Kizek 2023	D6 Measurement of outcomes	Moderate	Success defined by explicit criteria (no pain, mobility, periapical pathology) but assessed by unblinded clinicians; radiological ankylosis judgement unblinded.
Kizek 2023	D7 Selection of the reported result	Moderate	No protocol or registration recorded; results presented descriptively without confidence intervals or tests, allowing selective emphasis.
Kizek 2023	Overall	Serious	Overall judgement equals the most severe domain judgement (ROBINS-I convention).
Chen 2026	D1 Confounding	Moderate	Non-random allocation; baseline comparability of the two arms (age, donor/recipient site, bone quality) is not recorded in the extraction log; consecutive enrolment reduces but does not remove confounding.
Chen 2026	D2 Selection of participants	Moderate	Consecutive enrolment stated; method of assigning patients to guided versus freehand arms not recorded.
Chen 2026	D3 Classification of interventions	Low	Intervention arms clearly defined by workflow.
Chen 2026	D4 Deviations from intended interventions	Low	Single board-certified surgeon performed all procedures; no deviations recorded.
Chen 2026	D5 Missing data	Low	All 24 participants analysed (one immediate postoperative CBCT each).
Chen 2026	D6 Measurement of outcomes	Low	Outcomes are software-based CBCT superimposition deviations (BLAD, CDD, DSC), largely objective; assessor blinding not recorded but measurement error is unlikely to differ by arm.
Chen 2026	D7 Selection of the reported result	Low	Prospectively registered; primary outcome (BLAD) prespecified; all listed deviation metrics reported including non-significant ones.
Chen 2026	Overall	Moderate	Overall judgement equals the most severe domain judgement (ROBINS-I convention).
Keranmu 2021	D1 Confounding	Moderate	Allocation method not reported (randomisation not stated); baseline comparability of arms not recorded in the extraction log.
Keranmu 2021	D2 Selection of participants	Moderate	Recruitment from one hospital department; how patients entered each arm is not recorded.
Keranmu 2021	D3 Classification of interventions	Low	Intervention (CGF application) clearly defined.
Keranmu 2021	D4 Deviations from intended interventions	Low	Same replica-guided surgical protocol in both arms; no deviations recorded.
Keranmu 2021	D5 Missing data	Low	51 of 52 evaluated (one loss to follow-up).
Keranmu 2021	D6 Measurement of outcomes	Moderate	Pain (VAS) is patient-reported without blinding; periapical healing and success judged by unblinded clinicians. Internal inconsistency in reported healing percentages (23/26 reported as 88%; 3 reported as 22%).
Keranmu 2021	D7 Selection of the reported result	Moderate	No registration or protocol recorded; multiple outcomes with no stated primary; success difference reported as non-significant without effect estimate.
Keranmu 2021	Overall	Moderate	Overall judgement equals the most severe domain judgement (ROBINS-I convention).
Hou 2025 [23] (RoB 2)	D1 Randomisation process	Some concerns	Random number table stated; allocation concealment not described; baseline table by group verified
Hou 2025 [23] (RoB 2)	D2 Deviations from intended interventions	Low	Single surgeon applied allocated model; no crossover reported

Hou 2025 [23] (RoB 2)	D3 Missing outcome data	Some concerns	117 enrolled vs 116 analysed; no flow diagram
Hou 2025 [23] (RoB 2)	D4 Measurement of the outcome	Low / Some concerns	Non-operating surgeon recorded outcomes; blinding to group not stated; re-preparation count observable intra-operatively
Hou 2025 [23] (RoB 2)	D5 Selection of the reported result	High (verified)	No registration or protocol; abstract claims shorter operation period and reduced trauma without data; tables internally inconsistent
Hou 2025 [23] (RoB 2)	Overall	High / Some concerns (assessors to decide)	Driven by D5
Li 2025 [44]	D1 Confounding	Serious	Operator enrolled and assigned participants; baseline comparability tables exist (verified) but allocation open to indication bias
Li 2025 [44]	D2 Selection of participants	Serious	Patients with template fractures during surgery excluded post hoc from the guided arm
Li 2025 [44]	D3 Classification of interventions	Low	Interventions well defined and applied prospectively
Li 2025 [44]	D4 Deviations from intended interventions	Moderate	Same surgeon; co-interventions (PRF, trephine bone) differ between arms
Li 2025 [44]	D5 Missing data	Moderate	4/30 followed by telephone only
Li 2025 [44]	D6 Measurement of outcomes	Moderate	Accuracy objective (CBCT); clinical success assessed unblinded
Li 2025 [44]	D7 Selection of the reported result	Moderate	Registered ChiCTR2300074646 – compare outcomes with registry; EAT not reported
Xia 2024 [45]	D1 Confounding	Serious	Replica use likely era-dependent; ARR, RCT timing, age adjusted in Cox model but surgeon experience/period not
Xia 2024 [45]	D2 Selection of participants	Moderate	Retrospective records; inclusion criteria stated (verified)
Xia 2024 [45]	D3 Classification of interventions	Low	Replica use recorded in operative notes (verified)
Xia 2024 [45]	D4 Deviations from intended interventions	Moderate	ARR performed in subset; co-interventions differ
Xia 2024 [45]	D5 Missing data	Moderate	Follow-up SD 2 y; censoring handled by Cox; losses verified
Xia 2024 [45]	D6 Measurement of outcomes	Moderate	Record-based; assessors not blinded to replica use
Xia 2024 [45]	D7 Selection of the reported result	Low	Pre-specified prognostic analysis; all factors reported
EzEldeen 2019 [53]	D1 Confounding	Serious	Conventional group treated earlier (verified dates); surgeon experience and imaging era confounded with intervention
EzEldeen 2019 [53]	D2 Selection of participants	Moderate	Inclusion criteria stated; matching not described
EzEldeen 2019 [53]	D3 Classification of interventions	Low	Clear protocols
EzEldeen 2019 [53]	D4 Deviations from intended interventions	Low	Same surgical team
EzEldeen 2019 [53]	D5 Missing data	Moderate	Follow-up 1.1–10.4 y; censoring described (verified)
EzEldeen 2019 [53]	D6 Measurement of outcomes	Moderate	Clinical/radiographic assessment unblinded; 3D analysis objective
EzEldeen 2019 [53]	D7 Selection of the reported result	Moderate	Registered NCT02464202 – compare; 'P > 0.005' reporting
Shahbazian 2013 [57]	D1 Confounding	Serious	Historical control group
Shahbazian 2013 [57]	D2 Selection of participants	Serious	Selection of historical controls not described
Shahbazian 2013 [57]	D3 Classification of interventions	Low	Clear
Shahbazian 2013 [57]	D4 Deviations from intended interventions	Moderate	Verified
Shahbazian 2013 [57]	D5 Missing data	Low	All 48 teeth reported at 1 y (verified)
Shahbazian 2013 [57]	D6 Measurement of outcomes	Serious	Operative times given as ranges; unblinded

Shahbazian 2013 [57]	D7 Selection of the reported result	Moderate	No statistical comparison of operative outcomes

Supplementary Table S15. JBI case-series checklist item-level judgements and supporting statements for the sixteen single-arm or non-comparative studies.

Study	Item	Judgement	Support for judgement (from extracted data)
Salviati 2026	Q1 Clear criteria for inclusion	Yes	Eligibility 18-32 years with fully developed third-molar donors stated.
Salviati 2026	Q2 Condition measured in a standard, reliable way	Yes	CBCT plus intraoral scan with defined segmentation and superimposition protocol.
Salviati 2026	Q3 Valid methods used to identify the condition	Yes	Indication for autotransplantation and donor selection defined clinically and radiographically.
Salviati 2026	Q4 Consecutive inclusion of participants	Unclear	Prospective enrolment, but consecutive recruitment is not stated in the extraction log.
Salviati 2026	Q5 Complete inclusion of participants	Yes	12 enrolled, 2 deferred for orthodontic reasons and explained; all 10 treated patients followed to 18 months.
Salviati 2026	Q6 Clear reporting of demographics	Yes	Mean age with SD reported; sex distribution not recorded in the log but age and donor type given per patient.
Salviati 2026	Q7 Clear reporting of clinical information	Yes	Donor tooth identity, root morphology, surgical times and endodontic events reported per case.
Salviati 2026	Q8 Outcomes or follow-up results clearly reported	Yes	Accuracy, operative and clinical outcomes reported at scheduled visits to 18 months; survival not expressed as a percentage but all transplants accounted for.
Salviati 2026	Q9 Clear reporting of presenting site(s)/clinic(s) demographic information	Yes	Single university oral surgery clinic, Catanzaro, Italy.
Salviati 2026	Q10 Appropriate statistical analysis	Yes	Descriptive statistics (mean, SD) appropriate for a 10-patient accuracy series.
Salviati 2026	Overall appraisal	Include - low risk of bias	Overall appraisal is a narrative judgement weighting items Q4, Q5 and Q8 (selection and outcome completeness) most heavily; JBI does not prescribe a numerical cut-off.
Hadji 2024	Q1 Clear criteria for inclusion	Yes	Patients treated without a 3D replica were explicitly excluded; replica use required.
Hadji 2024	Q2 Condition measured in a standard, reliable way	Yes	Standard clinical and radiographic assessment at defined intervals (1, 3, 6 months, then annually).
Hadji 2024	Q3 Valid methods used to identify the condition	Yes	Indications and donor/recipient sites documented.
Hadji 2024	Q4 Consecutive inclusion of participants	Yes	Consecutive patients stated.
Hadji 2024	Q5 Complete inclusion of participants	Unclear	4 of 26 transplants lost to follow-up (15%); reasons not recorded in the log.
Hadji 2024	Q6 Clear reporting of demographics	Unclear	Median age reported; sex distribution not recorded in the extraction log.
Hadji 2024	Q7 Clear reporting of clinical information	Yes	Donor eruption status, apex maturity, endodontic treatment and extra-alveolar time reported.
Hadji 2024	Q8 Outcomes or follow-up results clearly reported	Yes	Survival, success, resorption, ankylosis, bone level and satisfaction reported with median observation periods.
Hadji 2024	Q9 Clear reporting of presenting site(s)/clinic(s) demographic information	Yes	Rennes Dental University Hospital, France.
Hadji 2024	Q10 Appropriate statistical analysis	Yes	Survival reported over median observation period; descriptive analysis appropriate.
Hadji 2024	Overall appraisal	Include - low to moderate risk of bias	Overall appraisal is a narrative judgement weighting items Q4, Q5 and Q8 (selection and outcome completeness) most heavily; JBI does not prescribe a numerical cut-off.
Singh 2024	Q1 Clear criteria for inclusion	Unclear	Inclusion and exclusion criteria are not recorded in the extraction log.
Singh 2024	Q2 Condition measured in a standard, reliable way	Yes	CAD/CAM replica workflow described; imaging modality stated.

Singh 2024	Q3 Valid methods used to identify the condition	Yes	Donor and recipient types documented by tooth category.
Singh 2024	Q4 Consecutive inclusion of participants	Unclear	Recruitment method (consecutive or otherwise) not recorded.
Singh 2024	Q5 Complete inclusion of participants	No	The paper reports 200 transplanted teeth in one place and 160 cases in another; the discrepancy is unexplained.
Singh 2024	Q6 Clear reporting of demographics	Unclear	Age reported by donor tooth type; sex and overall demographics not recorded in the log.
Singh 2024	Q7 Clear reporting of clinical information	Yes	Donor type, jaw, root-development stage and setting (operating room vs outpatient) reported.
Singh 2024	Q8 Outcomes or follow-up results clearly reported	Unclear	Intraoperative outcomes (extra-alveolar time, fitting attempts) reported, but there is no postoperative follow-up and the case-count inconsistency affects denominators.
Singh 2024	Q9 Clear reporting of presenting site(s)/clinic(s) demographic information	No	Country and participating centres not reported.
Singh 2024	Q10 Appropriate statistical analysis	Yes	Descriptive statistics appropriate for the intraoperative outcomes reported.
Singh 2024	Overall appraisal	Include with caution - high risk of bias	Overall appraisal is a narrative judgement weighting items Q4, Q5 and Q8 (selection and outcome completeness) most heavily; JBI does not prescribe a numerical cut-off.
Han 2022	Q1 Clear criteria for inclusion	Yes	Consecutive autotransplantation cases with CAD-based guide and model in all cases.
Han 2022	Q2 Condition measured in a standard, reliable way	Yes	CBCT-based planning and standardised intraoperative measurements (socket preparation time, extra-alveolar time, positioning trials).
Han 2022	Q3 Valid methods used to identify the condition	Yes	Third-molar donors with defined recipient indications.
Han 2022	Q4 Consecutive inclusion of participants	Yes	41 consecutive cases stated.
Han 2022	Q5 Complete inclusion of participants	Yes	All 41 cases reported, including the extracted failure.
Han 2022	Q6 Clear reporting of demographics	Yes	Age (mean, SD, range) and sex reported.
Han 2022	Q7 Clear reporting of clinical information	Yes	Apex maturity, endodontic treatment timing and complications reported.
Han 2022	Q8 Outcomes or follow-up results clearly reported	Yes	Periodontal healing at 6 months, resorption, periapical periodontitis and retention reported; 1-year follow-up stated.
Han 2022	Q9 Clear reporting of presenting site(s)/clinic(s) demographic information	Yes	Hospital of Stomatology, Wuhan University, China.
Han 2022	Q10 Appropriate statistical analysis	Yes	Between-group comparison of post hoc seated vs not-seated groups is exploratory; descriptive statistics appropriate.
Han 2022	Overall appraisal	Include - low risk of bias	Overall appraisal is a narrative judgement weighting items Q4, Q5 and Q8 (selection and outcome completeness) most heavily; JBI does not prescribe a numerical cut-off.
Wu 2019	Q1 Clear criteria for inclusion	Yes	Mature impacted third molars transplanted to fresh molar sockets, defined period Sept 2016 - Aug 2017.
Wu 2019	Q2 Condition measured in a standard, reliable way	Yes	CBCT (voxel 0.15 mm) and standardised clinical assessment.
Wu 2019	Q3 Valid methods used to identify the condition	Yes	Indications documented.
Wu 2019	Q4 Consecutive inclusion of participants	Yes	Consecutive patients stated.
Wu 2019	Q5 Complete inclusion of participants	Yes	All 10 patients followed for 2 years.
Wu 2019	Q6 Clear reporting of demographics	Yes	Age (mean, SD, range) and sex reported.
Wu 2019	Q7 Clear reporting of clinical information	Yes	Donor and recipient sites, RCT, GBR use reported.
Wu 2019	Q8 Outcomes or follow-up results clearly reported	Yes	Success by Tsukiboshi criteria, probing depths, mobility, resorption and ankylosis at scheduled recalls; note the extra-oral time is reported as 1.35 min in Results and 1.65 min in the abstract.

Wu 2019	Q9 Clear reporting of presenting site(s)/clinic(s) demographic information	Yes	Affiliated Stomatological Hospital of Fujian Medical University, China.
Wu 2019	Q10 Appropriate statistical analysis	Yes	Descriptive statistics appropriate for n = 10; no inferential claims.
Wu 2019	Overall appraisal	Include - low risk of bias (small series)	Overall appraisal is a narrative judgement weighting items Q4, Q5 and Q8 (selection and outcome completeness) most heavily; JBI does not prescribe a numerical cut-off.
He 2018	Q1 Clear criteria for inclusion	Yes	Enrolment age 18-40 with fully developed third-molar donors; IRB approval recorded.
He 2018	Q2 Condition measured in a standard, reliable way	Yes	CBCT-based accuracy analysis with defined landmarks (occlusal and apical points).
He 2018	Q3 Valid methods used to identify the condition	Yes	Indications documented.
He 2018	Q4 Consecutive inclusion of participants	Unclear	Recruitment method not recorded in the log.
He 2018	Q5 Complete inclusion of participants	Yes	All 8 patients reported with individual follow-up durations.
He 2018	Q6 Clear reporting of demographics	Yes	Age (mean, SD, individual ages) and sex reported.
He 2018	Q7 Clear reporting of clinical information	Yes	Donor identity, endodontic treatment and fixation reported per patient.
He 2018	Q8 Outcomes or follow-up results clearly reported	Yes	Accuracy, stability, success (7/8), survival (8/8), ankylosis (1) and periodontal status reported.
He 2018	Q9 Clear reporting of presenting site(s)/clinic(s) demographic information	Yes	Peking University School and Hospital of Stomatology, China.
He 2018	Q10 Appropriate statistical analysis	Yes	Descriptive statistics (mean, SD, range) appropriate.
He 2018	Overall appraisal	Include - low risk of bias (small series)	Overall appraisal is a narrative judgement weighting items Q4, Q5 and Q8 (selection and outcome completeness) most heavily; JBI does not prescribe a numerical cut-off.
Lee SJ 2012	Q1 Clear criteria for inclusion	Unclear	Inclusion criteria for the clinical series are not recorded in the log.
Lee SJ 2012	Q2 Condition measured in a standard, reliable way	Yes	CT-based CARP model with a documented cadaver validation of dimensional accuracy.
Lee SJ 2012	Q3 Valid methods used to identify the condition	Yes	Wisdom-tooth donors with prior endodontic treatment documented.
Lee SJ 2012	Q4 Consecutive inclusion of participants	Unclear	Recruitment method not recorded.
Lee SJ 2012	Q5 Complete inclusion of participants	No	Methods state 182 transplants but results refer to 251 cases; the discrepancy is unexplained and affects all denominators.
Lee SJ 2012	Q6 Clear reporting of demographics	Yes	Age (mean, range) and sex reported.
Lee SJ 2012	Q7 Clear reporting of clinical information	Yes	Endodontic management (intra-oral vs extra-oral) and donor type reported.
Lee SJ 2012	Q8 Outcomes or follow-up results clearly reported	Unclear	Extra-oral time and root-to-bone distance reported, but follow-up duration is not reported and the extra-oral time figures differ between Results and Discussion; root resorption (4/251) is reported without a follow-up period.
Lee SJ 2012	Q9 Clear reporting of presenting site(s)/clinic(s) demographic information	Yes	Dental Hospital of Yonsei University, South Korea.
Lee SJ 2012	Q10 Appropriate statistical analysis	Unclear	Descriptive only; no variance estimates recorded for the clinical outcomes (cadaver experiment excluded from this appraisal).
Lee SJ 2012	Overall appraisal	Include with caution - high risk of bias	Overall appraisal is a narrative judgement weighting items Q4, Q5 and Q8 (selection and outcome completeness) most heavily; JBI does not prescribe a numerical cut-off.

Pedrinaci 2024 [46]	Q1 Clear criteria for inclusion	Yes	
Pedrinaci 2024 [46]	Q2 Condition measured in a standard, reliable way	Yes	
Pedrinaci 2024 [46]	Q3 Valid methods used to identify the condition	Yes	
Pedrinaci 2024 [46]	Q4 Consecutive inclusion of participants	Yes (prospective, consecutive stated – verified)	
Pedrinaci 2024 [46]	Q5 Complete inclusion of participants	Yes	
Pedrinaci 2024 [46]	Q6 Clear reporting of demographics	Yes	
Pedrinaci 2024 [46]	Q7 Clear reporting of clinical information	Yes	
Pedrinaci 2024 [46]	Q8 Outcomes or follow-up results clearly reported	Yes (but 91.2% ≠ 12/13)	
Pedrinaci 2024 [46]	Q9 Clear reporting of site/clinic demographics	Yes	
Pedrinaci 2024 [46]	Q10 Appropriate statistical analysis	Yes	
Zhang 2024 [47]	Q1 Clear criteria for inclusion	Yes	
Zhang 2024 [47]	Q2 Condition measured in a standard, reliable way	Yes (Dolphin superimposition)	
Zhang 2024 [47]	Q3 Valid methods used to identify the condition	Yes	
Zhang 2024 [47]	Q4 Consecutive inclusion of participants	Unclear	
Zhang 2024 [47]	Q5 Complete inclusion of participants	Unclear	
Zhang 2024 [47]	Q6 Clear reporting of demographics	Partial (age verified)	
Zhang 2024 [47]	Q7 Clear reporting of clinical information	Yes	
Zhang 2024 [47]	Q8 Outcomes or follow-up results clearly reported	No (clinical outcomes narrative only)	
Zhang 2024 [47]	Q9 Clear reporting of site/clinic demographics	Yes	
Zhang 2024 [47]	Q10 Appropriate statistical analysis	Yes	
Ainiwaer 2024 [48]	Q1 Clear criteria for inclusion	Yes	
Ainiwaer 2024 [48]	Q2 Condition measured in a standard, reliable way	Yes	
Ainiwaer 2024 [48]	Q3 Valid methods used to identify the condition	Yes	
Ainiwaer 2024 [48]	Q4 Consecutive inclusion of participants	Unclear (retrospective)	
Ainiwaer 2024 [48]	Q5 Complete inclusion of participants	Yes	

Ainiwaer 2024 [48]	Q6 Clear reporting of demographics	Yes	
Ainiwaer 2024 [48]	Q7 Clear reporting of clinical information	Yes	
Ainiwaer 2024 [48]	Q8 Outcomes or follow-up results clearly reported	Yes	
Ainiwaer 2024 [48]	Q9 Clear reporting of site/clinic demographics	Yes	
Ainiwaer 2024 [48]	Q10 Appropriate statistical analysis	Yes	
Lucas-Taulé 2021 [49]	Q1 Clear criteria for inclusion	Yes	
Lucas-Taulé 2021 [49]	Q2 Condition measured in a standard, reliable way	Yes	
Lucas-Taulé 2021 [49]	Q3 Valid methods used to identify the condition	Yes	
Lucas-Taulé 2021 [49]	Q4 Consecutive inclusion of participants	No (selected: cases without CARP/<1 y/plate damage excluded)	
Lucas-Taulé 2021 [49]	Q5 Complete inclusion of participants	No	
Lucas-Taulé 2021 [49]	Q6 Clear reporting of demographics	Yes	
Lucas-Taulé 2021 [49]	Q7 Clear reporting of clinical information	Yes	
Lucas-Taulé 2021 [49]	Q8 Outcomes or follow-up results clearly reported	Yes	
Lucas-Taulé 2021 [49]	Q9 Clear reporting of site/clinic demographics	Yes	
Lucas-Taulé 2021 [49]	Q10 Appropriate statistical analysis	Yes	
Erdem 2020 [50]	Q1 Clear criteria for inclusion	Yes	
Erdem 2020 [50]	Q2 Condition measured in a standard, reliable way	Yes	
Erdem 2020 [50]	Q3 Valid methods used to identify the condition	Yes	
Erdem 2020 [50]	Q4 Consecutive inclusion of participants	Unclear	
Erdem 2020 [50]	Q5 Complete inclusion of participants	Yes	
Erdem 2020 [50]	Q6 Clear reporting of demographics	Yes	
Erdem 2020 [50]	Q7 Clear reporting of clinical information	Yes	
Erdem 2020 [50]	Q8 Outcomes or follow-up results clearly reported	Yes	

Erdem 2020 [50]	Q9 Clear reporting of site/clinic demographics	Yes	
Erdem 2020 [50]	Q10 Appropriate statistical analysis	Not applicable (descriptive)	
Lucas-Taulé 2020 [20]	Q1 Clear criteria for inclusion	Yes	
Lucas-Taulé 2020 [20]	Q2 Condition measured in a standard, reliable way	Yes	
Lucas-Taulé 2020 [20]	Q3 Valid methods used to identify the condition	Yes	
Lucas-Taulé 2020 [20]	Q4 Consecutive inclusion of participants	Yes (consecutive)	
Lucas-Taulé 2020 [20]	Q5 Complete inclusion of participants	Yes	
Lucas-Taulé 2020 [20]	Q6 Clear reporting of demographics	Yes	
Lucas-Taulé 2020 [20]	Q7 Clear reporting of clinical information	Yes	
Lucas-Taulé 2020 [20]	Q8 Outcomes or follow-up results clearly reported	Yes	
Lucas-Taulé 2020 [20]	Q9 Clear reporting of site/clinic demographics	Yes	
Lucas-Taulé 2020 [20]	Q10 Appropriate statistical analysis	Not applicable	
Verweij 2019 [51]	Q1 Clear criteria for inclusion	Yes	
Verweij 2019 [51]	Q2 Condition measured in a standard, reliable way	Yes (timing method verified)	
Verweij 2019 [51]	Q3 Valid methods used to identify the condition	Yes	
Verweij 2019 [51]	Q4 Consecutive inclusion of participants	Yes (consecutive)	
Verweij 2019 [51]	Q5 Complete inclusion of participants	Yes	
Verweij 2019 [51]	Q6 Clear reporting of demographics	Yes	
Verweij 2019 [51]	Q7 Clear reporting of clinical information	Yes	
Verweij 2019 [51]	Q8 Outcomes or follow-up results clearly reported	Yes (operative only)	
Verweij 2019 [51]	Q9 Clear reporting of site/clinic demographics	Yes	
Verweij 2019 [51]	Q10 Appropriate statistical analysis	Yes (descriptive)	
Wu 2019 BJOMS [52]	Q1 Clear criteria for inclusion	Partial	
Wu 2019 BJOMS [52]	Q2 Condition measured in a standard, reliable way	Yes	

Wu 2019 BJOMS [52]	Q3 Valid methods used to identify the condition	Yes	
Wu 2019 BJOMS [52]	Q4 Consecutive inclusion of participants	Unclear	
Wu 2019 BJOMS [52]	Q5 Complete inclusion of participants	Unclear	
Wu 2019 BJOMS [52]	Q6 Clear reporting of demographics	Partial	
Wu 2019 BJOMS [52]	Q7 Clear reporting of clinical information	Yes	
Wu 2019 BJOMS [52]	Q8 Outcomes or follow-up results clearly reported	Yes	
Wu 2019 BJOMS [52]	Q9 Clear reporting of site/clinic demographics	Partial	
Wu 2019 BJOMS [52]	Q10 Appropriate statistical analysis	Not applicable	
Tong 2021 [54]	Q1 Clear criteria for inclusion	Partial	
Tong 2021 [54]	Q2 Condition measured in a standard, reliable way	No (EAT not measured numerically)	
Tong 2021 [54]	Q3 Valid methods used to identify the condition	Yes	
Tong 2021 [54]	Q4 Consecutive inclusion of participants	Unclear	
Tong 2021 [54]	Q5 Complete inclusion of participants	Unclear	
Tong 2021 [54]	Q6 Clear reporting of demographics	Yes (age, teeth)	
Tong 2021 [54]	Q7 Clear reporting of clinical information	Partial	
Tong 2021 [54]	Q8 Outcomes or follow-up results clearly reported	No (no numeric outcomes)	
Tong 2021 [54]	Q9 Clear reporting of site/clinic demographics	Yes	
Tong 2021 [54]	Q10 Appropriate statistical analysis	Not applicable	
Kim 2005 [58]	Q1 Clear criteria for inclusion	Partial	
Kim 2005 [58]	Q2 Condition measured in a standard, reliable way	Yes	
Kim 2005 [58]	Q3 Valid methods used to identify the condition	Yes	
Kim 2005 [58]	Q4 Consecutive inclusion of participants	Unclear	
Kim 2005 [58]	Q5 Complete inclusion of participants	No (143/182 with first follow-up)	
Kim 2005 [58]	Q6 Clear reporting of demographics	Partial	

Kim 2005 [58]	Q7 Clear reporting of clinical information	Partial	
Kim 2005 [58]	Q8 Outcomes or follow-up results clearly reported	Partial	
Kim 2005 [58]	Q9 Clear reporting of site/clinic demographics	Yes	
Kim 2005 [58]	Q10 Appropriate statistical analysis	Yes	
Jang 2016 [4]	Q1 Clear criteria for inclusion	Yes	
Jang 2016 [4]	Q2 Condition measured in a standard, reliable way	Yes	
Jang 2016 [4]	Q3 Valid methods used to identify the condition	Yes	
Jang 2016 [4]	Q4 Consecutive inclusion of participants	Yes (all records 2001–2010)	
Jang 2016 [4]	Q5 Complete inclusion of participants	Yes (incomplete records excluded)	
Jang 2016 [4]	Q6 Clear reporting of demographics	Yes	
Jang 2016 [4]	Q7 Clear reporting of clinical information	Yes	
Jang 2016 [4]	Q8 Outcomes or follow-up results clearly reported	Yes	
Jang 2016 [4]	Q9 Clear reporting of site/clinic demographics	Yes	
Jang 2016 [4]	Q10 Appropriate statistical analysis	Yes (KM/Cox)	
Lee 2001 [17]	Q1 Clear criteria for inclusion	Partial	
Lee 2001 [17]	Q2 Condition measured in a standard, reliable way	Yes	
Lee 2001 [17]	Q3 Valid methods used to identify the condition	Yes	
Lee 2001 [17]	Q4 Consecutive inclusion of participants	Unclear	
Lee 2001 [17]	Q5 Complete inclusion of participants	No (2 failures excluded)	
Lee 2001 [17]	Q6 Clear reporting of demographics	Yes	
Lee 2001 [17]	Q7 Clear reporting of clinical information	Partial	
Lee 2001 [17]	Q8 Outcomes or follow-up results clearly reported	Partial	
Lee 2001 [17]	Q9 Clear reporting of site/clinic demographics	Yes	
Lee 2001 [17]	Q10 Appropriate statistical analysis	Not applicable	

Data extraction

Supplementary Table S16. Data extraction for the 25 included studies and their secondary reports (items not shown in Table 1 of the main text).

First author, year	Setting	Comparator / control	Digital guidance modality	Planning software / hardware	Donor tooth type / recipient site	Apex maturity	Primary outcomes	Secondary outcomes	Main results	Funding	Conflict of interest
Salviati 2026	University hospital - Oral Surgery Clinic, Department of Dentistry, Magna Graecia University of Catanzaro	None (single-arm; no control group)	Full digital workflow: CBCT + intraoral scan, AI/automatic segmentation, virtual autotransplantation planning with virtual subtraction of recipient bone, customised root-based 3D-printed surgical guides plus 3D-printed donor tooth replica	3D Slicer with DentalSegmentator extension (segmentation, registration, ModelToModelDistance, AQ3DC); RealGuide (3Diemme) for planning; FormLabs 3B printer with Surgical Guide resin	Erupted third molars: tooth 1.8 single-rooted (n=4), 4.8 multi-rooted (n=4), 2.8 multi-rooted (n=2)	Mature/closed apex - "All donor teeth exhibited complete root development"; "fully developed roots of the third molars"	Spatial deviation between planned and post-surgical donor tooth position (surgical accuracy)	Surgical time, extra-alveolar time, mobility, percussion sensitivity, pulp vitality, probing depth, tooth colour, masticatory function	Mean surface deviation 0.63 ± 0.45 mm (tooth) and 0.17 ± 0.06 mm (alveolar bone). 3D linear displacement 2.04 ± 1.24 mm coronal and 1.36 ± 1.00 mm apical; all axis-wise linear deviations <2 mm. Angular: yaw −6.06 ± 10.84 deg, pitch 1.86 ± 2.35 deg, roll −2.68 ± 5.8 deg. Mean extra-alveolar time 7 ± 19 s; mean surgical time 26.7 ± 12.1 min. 2 of 10 donor teeth required post-transplant endodontic treatment for periapical lesions. At 18 months: all transplants stable, physiological probing depths, no pathological mobility, no radiographic signs of resorption or periapical alteration; all non-endodontically treated teeth responded positively to vitality testing. Survival/success percentages not reported numerically.	Not reported	Not reported
Chen 2026	Single centre, single board-certified oral and maxillofacial surgeon (Guangzhou Medical University affiliated setting)	Alternative digital workflow - freehand socket preparation using the 3D-printed donor replica only (n=12) vs dual-template guided (n=12)	AI-driven CBCT segmentation, virtual planning, dual 3D-printed static templates (socket-margin guide plus metal-sleeved socket-preparation guide) and 3D-printed donor tooth replica	3D Slicer v5.7.0 with DentalSegmentator (AI segmentation); Mimics Research 21.0; PlastyCAD v1.7 (guide design); 3-matic 16.0; Geomagic Control X 2021 (measurement); 3Dme AI registration; Piocreat D158 DLP printer	Non-functional third molars transplanted to a single posterior recipient site (molar or premolar) in the same jaw; example given donor #38 to site #46	Not reported	Buccolingual angular deviation (BLAD) between planned and achieved donor tooth position	Centre deviation distance (CDD), index-point deviations at crown and root (IPDD-C, IPDD-R), long-axis (LAD), mesiodistal (MDAD) and rotational (RAD) deviations, Dice similarity coefficient (DSC)	Guided vs freehand: BLAD 2.76 ± 2.31 deg vs 8.61 ± 4.55 deg (p=.001, d=−1.62); RAD 4.37 ± 2.11 deg vs 16.05 ± 10.50 deg (p=.003, d=−1.54); CDD 1.00 ± 0.33 mm vs 2.00 ± 1.30 mm (p=.023); IPDD-C 1.29 ± 0.41 mm vs 2.97 ± 2.55 mm (p=.045); IPDD-R 2.04 ± 0.98 mm vs 4.02 ± 2.67 mm (p=.030); DSC 83.50 ± 4.74% vs 69.50 ± 14.02% (p=.006). LAD non-significant (p=.076); MDAD unchanged (p=.947). No survival, resorption, ankylosis or extra-alveolar time values reported.	Funding statement present but funder name not rendered in the retrieved full text ("This work was supported by ().")	The authors declare that they have no potential conflict of interest.
Hadji 2024	University hospital - Rennes Dental University Hospital	None (single-arm; one-stage/no-replica patients excluded by design)	CBCT-based CAD/CAM 3D-printed donor tooth replica used to prepare the recipient site (two-stage technique with double periodontal ligament stimulation)	Blue Sky Plan (segmentation); Meshmixer (Autodesk) for artefact removal; 3D printing in biocompatible resin	Second premolar most frequent donor, maxillary central incisor most frequent recipient; 14 donor teeth fully erupted, 3 partially erupted, 5 impacted	Mixed - 9 of 22 transplants immature at surgery; 13 had complete root length and underwent root canal treatment	Survival rate	Clinical and radiographic success, PDL healing, root resorption, ankylosis, pulp canal obliteration, root development, bone level change, extra-alveolar time, patient satisfaction	Survival 95.5% (21/22) after median 23 months; one failure due to chronic periapical inflammation at 16 months. Success 90.5% (19/21) after median 25.5 months. No root resorption and no ankylosis in any examined transplant; favourable PDL healing in 95% of transplants. No measurable bone loss; 81% showed bone level growth. Pulp cavity obliteration observed in all examined immature teeth; 2 immature transplants required root canal treatment. Average extra-alveolar time under 1 minute except two cases (5 min	Not reported	Not reported

									and 3 min). 100% of patients reported complete satisfaction.		
Lejnieks 2025	University clinic - Institute of Stomatology, Riga Stradins University	Conventional freehand autotransplantation without CBCT or 3D replica (control n=22)	CBCT-based planning plus 3D-printed donor tooth replica used to adapt the recipient site	Not reported (CBCT i-CAT Next Generation, KaVo; Asiga Max UV printer; replica steam-sterilised 121 C)	Unerupted third molars transplanted to premolar, first or second molar sites	Immature - Moorrees stages 3-5 (root 1/2 to open apex); stage 3 in 38%/36%, stage 4 in 50%/27%, stage 5 in 13%/36% (3D/control)	Tooth mobility (sample size powered on this), bleeding on probing, periodontal pocket depth	Radiographic root development, pulp canal obliteration, periapical status, crown/root ratio change, external root resorption, total surgery time, extra-alveolar time, donor fitting attempts	100% survival at 1 year in both groups. At 12 months, 3D-replica vs control: mobility 0% vs 13.6%; bleeding 4.1% vs 13.6%; periodontal pockets 4.1% vs 13.6%. No external root resorption in 88% (3D) vs 59% (control), resorption in 13% vs 36% (p=0.057). Root development 79% vs 64% (p=0.3); periapical status p=0.7; crown/root ratio change p=0.4. Total surgery time 45 min (IQR 40-51) vs 60 min (IQR 50-70); extra-alveolar time 35 s (IQR 30-60) vs 55 s (IQR 30-83); single donor fitting in 63% vs 32%. In adjusted mixed models, group assignment was not a significant predictor of mobility, bleeding or pocket depth; operative time, Moorrees stage and donor fitting times were.	Not reported (funding acquisition listed under author contributions but no funding statement given)	The authors declare no conflicts of interest.
Singh 2024	Multicentre; 51 autotransplantations performed in the operating room and 101 in the outpatient department (OPD)	none (single-arm)	CAD/CAM-designed, 3D-printed cobalt-chrome donor tooth replica used intraoperatively to prepare and check the recipient socket	Not reported (CAD-CAM and 3D printing stated; software name not given)	146 premolars (120 patients), 48 molars (36 patients), 4 canines (4 patients), 2 supernumerary (1 patient); 184 mandibular, 16 maxillary	Root development 50-75% in 147 teeth, 75-90% in 30 teeth, >90% in 23 teeth; mostly open apex	Extra-alveolar time and number of attempts to fit the donor tooth (intraoperative feasibility)	Total procedure time; intraoperative difficulties related to imaging, patient cooperation and bone quality	Mean extra-alveolar time 51 ± 86 seconds overall (canine 72±41, molar 81±125, premolar 38±105, supernumerary 59±58 s); 97 teeth transplanted in under 1 min, 15 under 2 min, 30 under 3 min, 10 over 3 min; total procedure time approximately 30 min; immediate satisfactory fit with no extra attempt in 80%, 1 attempt 11% (22 teeth), 2 attempts 2% (4 teeth), 3 attempts 6% (12 teeth), >3 attempts 1% (2 teeth); no survival, success, resorption or ankylosis data reported	Nil ("Financial support and sponsorship : Nil")	There are no conflicts of interest
Hwang 2022	Chang Gung Memorial Hospital, Kaohsiung and Chia-Yi branches, dental department; all RP cases by one experienced oral and maxillofacial surgeon	Conventional freehand method using the extracted donor tooth itself as the template for recipient site preparation (13 patients)	CBCT-based segmentation with computer-aided rapid prototyping: fused filament fabrication 3D-printed donor tooth model, alginate negative, Orthoresin donor tooth replica used as an intraoperative surgical template	ITK-SNAP v3.6.0 (active contour segmentation, lower threshold 1400) for segmentation; Autodesk Meshmixer for mesh polishing; FlashForge Creator Pro printer with 1.75 mm polylactic acid	Mostly third molars as donors; recipient site most commonly the first molar, then the second molar	Adults (>=18 years) with already formed donor tooth roots (mature); continuous root formation observed in some RP cases that avoided RCT	Root canal treatment rate and transplant survival	Extra-alveolar time, procedure time, age, sex, fixation method	Survival rate 100% in both groups; RCT rate 92.3% conventional vs 59% RP in the abstract, with the Results text stating 50% of RP patients required RCT; after excluding all patients aged >40 years the RCT rate remained significantly lower in the RP group (50% vs 91%, p=0.048); mean extra-alveolar time 43 seconds in the RP group (reported as 46 seconds in the Discussion); age differed significantly between groups (28.8 ± 10 vs 21.6 ± 2.1, p=0.025) and was non-significant after excluding patients >40 years (p=0.082); no resorption or ankylosis rates reported	Not reported	Not reported
Han 2022	University stomatological hospital, Department of Oral Surgery	No freehand or external comparator; internal comparison of group where donor tooth could	CBCT-based virtual planning + 1:1 3D-printed donor tooth replica + 3D-printed surgical guide plate	Mimics Medical 20.0 and Materialise 3-matic 11.0; NewTom NNT for CBCT; Formlabs Form2 SLA printer	All donor teeth were third molars	Mostly closed apices; 4 cases had immature donor teeth (1 completed development with pulp	Alveolar socket preparation time, extra-alveolar time of donor tooth, number of positioning trials with donor tooth	Periodontal healing, external root resorption, periapical periodontitis, tooth retention/survival	Mean socket preparation time 12.73 ± 6.18 min (range 3-30); mean extra-alveolar time 5.56 ± 3.11 min (range 1-15), significantly less than 15 min (p<0.05); mean positioning trials 2.61 ± 1.00 (range 1-5). Unsuccessful-seating group 15.57 ± 6.14 min, 7.29 ± 2.57	Not reported in retrieved full text	Not reported in retrieved full text

		be seated successfully (20 cases) vs could not (21 cases)		(layer 0.05 mm), photopolymer resin		healing, 3 needed RCT for apical periodontitis)		, RCT requirement	min, 3.19 ± 0.75 trials vs successful group 9.75 ± 4.73 min, 3.75 ± 2.57 min, 2.00 ± 0.86 trials (all p<0.05). Periodontal healing in 38/41 cases at 6 months; 1 periapical periodontitis (delayed RCT), 1 external root resorption (incomplete RCT), 1 tooth extracted due to infection without timely RCT; no significant difference in retention rate between groups. RCT completed in 29 cases at 2-4 weeks; 6 cases had root-tip resection with iRoot BP due to root fracture during extraction.		
Wu 2019	University stomatological hospital, Department of Oral and Maxillofacial Surgery	None (single-arm); informal comparison of cases with vs without GBR	CBCT-based segmentation and 3D-printed resin donor tooth replica used to prepare the recipient socket; guided bone regeneration with autogenous bone + CGF membrane where needed	Materialize Proplan software; NewTom GiANO CBCT (voxel 0.150 mm); Vida (Envision TEC) 3D printer	Mature impacted third molars transplanted into fresh first or second molar extraction sockets	All mature with completely formed roots (closed apices); all received RCT	Success rate per Tsukiboshi criteria (clinical and radiographic)	Extra-oral time, probing depth, mobility, percussion, masticatory function, root resorption, ankylosis, marginal bone loss	100% success at 2 years; all patients met success criteria with no periodontal pocket, mobility, inflammation or root absorption. Average extra-oral time 1.35 min (Results section; abstract states 1.65 min), range 0-4 min; 3 donor teeth transplanted in under 1 min; 2 cases took 3.5 and 4 min due to replica error. At 4 weeks: probing depth >3 mm in 3 patients and >4 mm in 1 patient (all distal-buccal/lingual), 1 patient with slight percussion pain, 1 with slight sensitivity on chewing; 1 patient still >3 mm at 3 months. No bone loss exceeding one third of root length, no ankylosis and no root resorption on radiographs during the 2-year follow-up.	Not reported in retrieved full text	Not reported in retrieved full text
He 2018	University school and hospital of stomatology	None (single-arm); designed virtual position used as reference for accuracy	Computer-aided surgical simulation with 3D-printed Co-Cr donor tooth replica + local occlusal splint + individual surgical template + individual arch bar for fixation	Mimics 16.0 (Materialise); FreeForm Modeling Plus v11 with Phantom Desktop arm (Geomagic SensAble); Geomagic Studio for accuracy analysis; EOS M280 3D printer; NewTom VG CBCT (voxel 0.15 mm)	Third molars (18, 28, 38, 48) transplanted to first or second molar sites	All roots of donor teeth fully developed (mature); all required root canal treatment	Feasibility, linear accuracy (deviation of designed vs achieved position) and postoperative positional stability	Extra-alveolar time, success rate, 1-year survival, mobility, probing depth, ankylosis, root resorption, pulp vitality, planning time	Success rate 87.5% (7/8) with ankylosis in 1 patient; 1-year survival 100% (8/8). Extra-alveolar time under 3 min in all cases (immediate in 6 cases, 2 min 50 s and 2 min 40 s in 2 cases); 6 teeth fitted well, 2 needed additional socket preparation. Linear accuracy: occlusal point 1.43 ± 0.57 mm (range 0.69-2.08), apical point 1.77 ± 0.67 mm (range 0.72-2.32). Linear stability: occlusal point 0.66 ± 0.36 mm (0.30-1.33), apical point 0.67 ± 0.48 mm (0.21-1.59). Stability achieved at 3 or 6 months with no or <1 mm mobility; pocket depths 2-4 mm; no continuous bone loss or root resorption; all teeth needed RCT (no pulp vitality at 2 weeks). Virtual planning and guide design took about 4 h per patient.	Not reported in retrieved full text	Not reported in retrieved full text
Kizek 2023	Hospital/university department not further specified; all procedures performed by the same surgeon	Conventional autotransplantation without replica (Group 2, 14 patients)	CBCT-based segmentation and DLP 3D-printed biocompatible donor tooth replica used intraoperatively to	Mimics V21 (Materialise, Leuven) for CBCT segmentation; PreForm (Formlabs) for print	Mainly ectopically impacted maxillary canines; second most frequent	Not reported systematically; the paper notes pulp vitality preservation applies to	Success rate at 1 year (no pain, no mobility, no periapical resorption or pathology)	Extra-alveolar time, ankylosis rate, need for endodontic treatment, failure rate	Success rate 90% with replica (Group 1) vs 71.4% without (Group 2); extra-alveolar time averaged 12 seconds with replica vs 5.5 minutes without; radiological signs of ankylosis in 3/14 Group 2 patients at 6 months post-op	Not reported	Not reported

			prepare, check and hold the neo-alveolus (angulation, depth, occlusal clearance)	preparation; EnvisionTEC Vida DLP printer with E-Guard material	indication was replacement of the first mandibular molar (usually with a wisdom tooth)	incompletely developed roots and that wisdom tooth apices are often still open			(none reported in Group 1); endodontic treatment required in 3 Group 1 patients and 4 Group 2 patients; no formal statistical comparison reported		
Keranmu 2021	Hospital department of oral surgery	Concentrated growth factor (CGF) vs no CGF; both arms used the 3D-printed replica, so no freehand comparator	CBCT-based 3D-printed resin donor tooth replica used to guide socket preparation (ethylene-oxide sterilised acrylic model)	MIMICS software; 3D printer (model not stated); CBCT (device not stated)	Mature third molars transplanted to fresh mandibular first or second molar sockets with chronic periapical lesions	All donor teeth were third molars with closed apices (mature)	Success rate (Andreasen criteria); radiographic healing of periapical lesions	Postoperative pain (VAS days 1/3/7), initial stability, probing depth, bleeding on probing, clinical attachment loss, mobility, root resorption, ankylosis, extra-oral time, surgery time	Success 100% (25/25) in CGF group vs 92.3% in control group (difference not significant, $p>0.05$). Average surgery time 39 min (± 7.8); average extra-oral time 42 s (± 10.2), all donor teeth transplanted within 60 s of extraction. CGF group: 23/26 (reported as 88%) periapical lesions healed completely within 3 months, remaining 3 (reported as 22%) within 6 months. Control group: 9 (35%) healed within 3 months, 17 (65%) within 6 months. VAS lower in CGF group on days 1 and 3 ($p<0.05$); initial stability better in CGF group ($p<0.05$). No root resorption, ankylosis, abnormal mobility or periodontal pocket in CGF group over 2 years; 2 failures in control group (1 probing depth >3 mm distal-buccal/lingual, 1 external root resorption at 2 years after delayed RCT).	Not reported in retrieved full text	Not reported in retrieved full text
Lee SJ 2012	University dental hospital	None (single-arm clinical series); cadaver arm compares real tooth vs 3D CT image model vs CARP model	CT-based computer-aided rapid prototyping (CARP) life-sized duplicate donor tooth model plus RP artificial jaw model for preoperative rehearsal of recipient site contouring	CT Highspeed Advantage with DentaScan (GE Medical Systems, slice thickness 1 mm); V-works / V-works 4.0 visualization software (Cybermed, Seoul); rapid prototyping machine, starch/resin model material	Wisdom teeth (third molars) used as donors	Fully developed roots; root canal treatment performed before extraction in most cases (168 intra-oral, 9 extra-oral endodontics)	Extra-oral time of the donor tooth; radiographic distance between transplanted root surface and alveolar bone; dimensional accuracy of the CARP model (cadaver experiment)	Root resorption	Extra-oral time averaged 7 min 25 s (range immediate to 25 min) without extra-oral RCT and 9 min 15 s with extra-oral RCT (Discussion instead states an overall mean of 7 min 45 s, 7 min 10 s in the 168 intra-oral endodontic patients and 15 min 20 s in the 9 extra-oral endodontic patients); 80.8% under 10 min, 11.3% 10-15 min, 7.9% over 15 min. Mean root-to-alveolar bone distance 1.17 mm mesio-cervical, 1.35 mm mesio-apical, 0.98 mm disto-cervical, 1.26 mm disto-apical. Root resorption observed in 4 of 251 cases (1.6%). Cadaver accuracy: mean absolute difference 0.199 mm real teeth vs 3D CT image, 0.169 mm 3D CT image vs CARP model, 0.291 mm real teeth vs CARP model; 3D CT image 0.149 mm smaller than real teeth and CARP model 0.216 mm smaller than real teeth.	Not reported in retrieved full text	Not reported in retrieved full text
Hou 2025 [23]	Stomatological Hospital, Fourth Military Medical University, Xi'an; single surgeon; Jan–Dec 2024	Universal (generic, population-derived) replica vs customised CBCT-printed replica –	Physical donor-tooth model used to prepare recipient socket (customised = patient-specific; universal = 12 standard variants)	Third molars → first/second molar sites (maxillary 50, mandibular 66)	Mature (adults 18–60)	Socket re-preparations after model use; initial stability; 6-mo healing	—	Customised model: fewer re-preparations ($P = .025/.042$); stability NS; 6-mo healing NS ($P = .327/.747$)	Shaanxi health research plan 2025YF-10; State Key Lab project	None declared	Ethics KQ-YJ-… (verified number); no trial registration stated

		alternative digital workflow				(success/retention/failure)					
Li 2025 [44]	Guangzhou Medical University hospital; Dec 2022–Jul 2023	Replica-only freehand preparation (n=15) vs fully guided system (n=15)	Replica + 3D-printed preparation (trephine), position and fixation guides	Molars; mature	Closed apex	Positional accuracy (plan vs CBCT); fitting attempts; socket time; clinical success ~14 mo	Mastication score; PD; PDL continuity	Accuracy better with guides; attempts/time NS; success 100% vs 70%	Guangzhou Medical University research project	None declared	ChiCTR2300074646; ethics LCYJ20230321; appendix states no random sequence
Xia 2024 [45]	Stomatology Hospital, Zhejiang University School of Stomatology, Hangzhou; retrospective	No replica (n=68 teeth) vs replica (n=100)	Donor tooth replica (SLA) guiding socket preparation	Third molars → molar sites	Complete root formation	Success; survival; inflammatory root resorption (Cox regression)	Surgical time; extraoral time; prognostic factors (ARR, RCT timing, age)	Replica associated with higher success/survival, less IRR; shorter times; ARR harmful	Zhejiang Provincial grant 2021C03059	None reported	Institutional approval (verified)
Pedrinaci 2024 [46]	Graduate Periodontology, Complutense University Madrid; prospective	None; EMD applied in all	CBCT virtual planning; printed replica; multidrilling-axis guide	Closed-apex third molars → hopeless molar sites	Closed apex	Survival; success at 2 y	PD, CAL, bone levels, ridge dimensions, PROMs	Survival 100%; success 91.2% (reported); stable ridge	No financial support	None declared	PROCESS 2023; ethics verified
Zhang 2024 [47]	School & Hospital of Stomatology, Wuhan University; Dec 2020–Sep 2022; single surgeon	None (planned vs achieved position)	Replica + positional guide	29 single-root, 21 multi-root; 21 max / 29 mand; 11 anterior / 39 posterior	48/50 fully formed	Positional deviation (cervical, apical, angular)	Factors affecting deviation	Cervical 1.16 mm; apical 1.80 mm; 6.82°; larger apical deviation in multi-root	NSFC 81800995	None	Ethics verified
Ainiwaer 2024 [48]	First Affiliated Hospital, Xinjiang Medical University, Urumqi; Jan 2017–Jun 2022; single surgeon	Surgically created socket (48) vs fresh extraction socket (48); all replica-guided	3D-printed donor replica (Mimics)	Mature third molars; mainly mandibular recipients	Mature	Survival/success; complications	EAT, trials, operation time, occlusal contact, crown restoration	Success 95.8% both groups; EAT ~61 s; trials 2.4	Hospital youth project	None	Ethics verified
Lucas-Taulé 2021 [49]	Private clinic, Barcelona; 2015–2019; single surgeon + endodontist	None (cases without planning/CARP excluded)	Virtual planning + CARP replica	Third molars (24 max / 12 mand)	12 open / 24 closed	Survival; success (PPD ≤ 4, CAL ≤ 5, no progressive resorption)	PPD, CAL change, complications	Survival 97.2%; success 91.7% at 29 mo	Institutional	None	UIC CIR-ECL-2020-02
Erdem 2020 [50]	Marmara University School of Dentistry, Istanbul; retrospective	None	Chrome-cobalt CARP replica for socket preparation	Impacted third molars → mandibular first/second molar sites with periapical lesions	Immature	Success (clinical + radiographic) at ≥ 18 mo	Stability, BOP, pocket, vitality, lamina dura, lesion healing	12/12 successful; all vital; no resorption/ankylosis	NR	None	Informed consent; ethics verified
Lucas-Taulé 2020 [20]	Private office, Barcelona; Jan 2018–Oct 2019; single surgeon	None	CARP replica + milled multidrilling-axis guide (fully guided osteotomy)	6 premolars, 4 third molars; 8 immediate / 2 healed ridge	6 open / 4 closed	Success/survival ≥ 6 mo	Complications, PD, pulp status	10/10 success; 1 arrested IRR	Not stated	None declared	Verified
Verweij 2019 [51]	LUMC Leiden + Amphia Hospital Breda;	None	CBCT + CAD/CAM replica	73 premolars, 24 molars, 2 canines, 1 supernumerary	Mostly 50–90% root development	Extra-alveolar time; fitting attempts	Causes of intra-operative difficulty	EAT 49 s; immediate fit 82%	NR	None	Leiden ethics approval

	consecutive Sept 2015–Jan 2019										
Wu 2019 BJOMS [52]	Stomatological hospital (verified institution); 5 patients	None	CAD surgical + location templates + RP tooth model (ProPlan CMF)	verified (third molars → molar sites)	verified	Survival/healing 6 mo; template fit; accuracy (volume overlap)	Socket preparation time	5/5 survived; overlap 99.4%	NR	None	Ethics approved
EzEldeen 2019 [53]	University Hospitals Leuven; children	Conventional TAT with 2D planning (50 transplants/44 pts)	CBCT-guided planning; replica + guide (in-house printed)	Premolars → incisor/premolar sites in children	Immature	Survival; success (mean 4.5 y)	3D healing patterns (RV, RL, AFA, DWT); EAT; surgery time (narrative)	Survival 92 vs 84%; success 86 vs 78% (NS)	verified	verified	NCT02464202; ethics S55287
Tong 2021 [54]	Townsville Hospital, Queensland; GA; 3 surgeons	None	3D-printed replica + MED610 printed surgical guide	Premolars and third molars (donors 14, 15, 18, 28, 35, 38, 45, 48 → various)	14 immature / 1 closed	Fit at first attempt; EAT; 6-mo clinical/radiographic status	—	15/15 first-attempt fit; EAT < 30 s; all vital/stable at 6 mo	NR	None	Townsville HHS HREC
Lejnieks 2023 [55]	Riga Stradins University; Aug 2019–Sep 2022	Conventional (22) vs replica (24)	CBCT + replica	Immature third molars	Immature	EAT (primary)	Total surgery time; attempts	EAT NS; total time −13.4 min	EU Horizon 2020 857287	None	ISRCTN13563091
Xia 2020 [56]	Second Affiliated Hospital, Zhejiang University; single clinician	None	CARP replica	Mature third molars → first/second molar sites	Mature	Success 2 y; EAT; surgery time	VAS pain; cervical gap	100% success; EAT 2.5 min	Not stated	None	Verified
Verweij 2016 [19]	LUMC Leiden	None	Titanium replica as guide	Maxillary premolars	Optimal root development	EAT; attempts; procedure time	—	All < 1 min, immediate fit	NR	None	Verified
Shahbazian 2013 [57]	KU Leuven; children	Historical conventional controls (20 pts/24 teeth)	CBCT + stereolithographic replica + drill guides (SimPlant)	Premolars (22) + molars (2) per arm	Immature	Attempts; EAT; surgery time; complications at 1 y	—	Fewer attempts, shorter EAT, fewer failures (2 vs 6)	verified	verified	Informed consent; ethics verified
Kim 2005 [58]	Yonsei University College of Dentistry, Seoul	None	CARP replica	Mostly molars (verified)	verified	Healing category; failure; resorption; ankylosis	EAT; initial stability	Failure 4.5%; ankylosis 10.7%; EAT 7.58 min	NR	NR	Verified
Jang 2016 [4]	Yonsei University Dental Hospital; Jul 2001–Aug 2010	None	CARP models (protocol)	Complete root formation (verified types)	Mature	Survival (KM); IRR; ankylosis	Prognostic factors (Cox)	68.2% survival at 12 y	verified	verified	Verified
Lee 2001 [17] / Lee 2004 [59]	Yonsei University	None	CT → CARP (or manual) replica; jaw model rehearsal	Mostly mandibular molars	Mature	EAT; root–bone gap	1-y clinical status (2001)	EAT 7.4 / 6.7 min; gap ~1 mm	NR	NR	NR

Reporting checklists

Supplementary Table S17. PRISMA 2020 checklist.

Section / topic	Item	Checklist item	Location where item is reported
Title	1	Identify the report as a systematic review	Title
Abstract	2	See PRISMA 2020 for Abstracts checklist	Abstract
Rationale	3	Describe the rationale for the review in the context of existing knowledge	Introduction, paragraphs 1–4

Objectives	4	Provide an explicit statement of the objective(s) or question(s)	Introduction, final paragraph
Eligibility criteria	5	Specify the inclusion and exclusion criteria and how studies were grouped for synthesis	Methods 2.2; 2.9
Information sources	6	Specify all databases, registers, websites, organisations, reference lists and other sources; date last searched	Methods 2.3
Search strategy	7	Present the full search strategies for all databases, registers and websites, including filters and limits	Methods 2.4; Supplementary Tables S1–S8
Selection process	8	Specify the methods used to decide whether a study met the inclusion criteria, including reviewers, independence and automation tools	Methods 2.5 (including retrieval routes and linking of multiple reports)
Data collection process	9	Specify the methods used to collect data from reports, including reviewers, independence and author contact	Methods 2.6
Data items	10a	List and define all outcomes for which data were sought	Methods 2.7
	10b	List and define all other variables for which data were sought	Methods 2.6
Study risk of bias assessment	11	Specify the methods used to assess risk of bias, including tools, reviewers and independence	Methods 2.8 (RoB 2, ROBINS-I, JBI); Supplementary Tables S13–S15
Effect measures	12	Specify for each outcome the effect measure(s) used	Methods 2.9 (narrative synthesis; outcomes as reported)
Synthesis methods	13a	Describe the processes used to decide which studies were eligible for each synthesis	Methods 2.9
	13b	Describe any methods required to prepare the data for presentation or synthesis	Methods 2.6, 2.9
	13c	Describe any methods used to tabulate or visually display results	Methods 2.9; Tables 1–3; Figures 2–3
	13d	Describe any methods used to synthesise results and rationale for the choice	Methods 2.9 (SWiM)
	13e	Describe any methods used to explore possible causes of heterogeneity	Methods 2.9 (grouping by design and modality)
	13f	Describe any sensitivity analyses	Not applicable (no meta-analysis)
Reporting bias assessment	14	Describe any methods used to assess risk of bias due to missing results	Methods 2.8 (ROBINS-I domain 7); Discussion 4.3
Certainty assessment	15	Describe any methods used to assess certainty in the body of evidence	Methods 2.9 (GRADE)
Study selection	16a	Describe the results of the search and selection process, ideally with a flow diagram	Results 3.1; Figure 1; Supplementary Tables S9–S10 (retrieval log); linked reports listed in Table 1
	16b	Cite studies that appeared to meet the inclusion criteria but were excluded, and explain why	Results 3.1; Supplementary Table S9 (n = 18)
Study characteristics	17	Cite each included study and present its characteristics	Results 3.2; Table 1 (25 studies; secondary reports listed beneath primary reports)
Risk of bias in studies	18	Present assessments of risk of bias for each included study	Results 3.7; Figure 3; Supplementary Tables S13–S15 (RoB 2, ROBINS-I, JBI)
Results of individual studies	19	For all outcomes, present for each study summary statistics and an effect estimate	Results 3.4–3.6; Table 2; Supplementary Table S12; Figure 3
Results of syntheses	20a	For each synthesis, briefly summarise the characteristics and risk of bias among contributing studies	Results 3.6–3.7
	20b	Present results of all statistical syntheses	Not applicable (narrative synthesis)
	20c	Present results of all investigations of possible causes of heterogeneity	Results 3.3–3.6 (by modality and design)
	20d	Present results of all sensitivity analyses	Not applicable
Reporting biases	21	Present assessments of risk of bias due to missing results	Results 3.7; Discussion 4.3
Certainty of evidence	22	Present assessments of certainty in the body of evidence for each outcome	Results 3.8; Table 3

Discussion	23a	Provide a general interpretation of the results in the context of other evidence	Discussion 4.1–4.2
	23b	Discuss any limitations of the evidence included in the review	Discussion 4.3
	23c	Discuss any limitations of the review processes used	Discussion 4.3
	23d	Discuss implications of the results for practice, policy and future research	Discussion 4.4
Registration and protocol	24a	Provide registration information for the review	Methods 2.1; Declarations
	24b	Indicate where the review protocol can be accessed	Methods 2.1
	24c	Describe and explain any amendments to information provided at registration or in the protocol	Methods 2.1
Support	25	Describe sources of financial or non-financial support and the role of funders	Declarations
Competing interests	26	Declare any competing interests of review authors	Declarations
Availability of data, code and other materials	27	Report which of the following are publicly available and where: data collection forms, extracted data, analytic code, other materials	Declarations; Supplementary Material

From Page MJ, et al. BMJ 2021;372:n71.

Supplementary Table S18. PRISMA-S checklist for literature searches.

**Section / topic**	**Item**	**Checklist item**	**Location**
Information sources and methods	1	Name each individual database searched, with the platform	Methods 2.3; S1–S8
	2	Name any study registries searched	Methods 2.3; S7
	3	Describe any online resources searched (e.g., Google Scholar)	Methods 2.3; S8
	4	Describe any citation searching	Methods 2.3; Results 3.1
	5	Describe any contact with authors or experts	Methods 2.6
	6	Describe any other methods	Not applicable
Search strategies	7	Include the full search strategy for each database	S1–S8
	8	State whether published search filters were used	Methods 2.4 (none)
	9	State whether prior work was used to develop the strategy	Methods 2.4
	10	Describe any limits (language, date, publication type) with justification	Methods 2.2, 2.4
	11	Describe how the master strategy was translated for other databases	Methods 2.4
	12	State the date each search was conducted or updated	Methods 2.3; S1–S8
	13	State whether the strategies were peer reviewed	PRESS peer review not performed
	14	State whether records were managed in a reference manager	Methods 2.5
Managing records	15	Describe deduplication methods	Methods 2.5
	16	Report the total number of records identified from each source	Figure 1; S1–S8

From Rethlefsen ML, et al. Syst Rev 2021;10:39.
